# Dual-channel mechano-phosphorescence: a combined locking effect with twisted molecular structures and robust interactions

**DOI:** 10.1038/s41377-024-01421-5

**Published:** 2024-04-08

**Authors:** Zongliang Xie, Zhu Mao, Hailan Wang, Yuxin Xiao, Xiayu Zhang, Tao Yu, Zhongfu An, Wei Huang

**Affiliations:** 1 Frontiers Science Center for Flexible Electronics, Xi’an Institute of Flexible Electronics & Xi’an Institute of Biomedical Materials and Engineering, Northwestern Polytechnical University, 127 West Youyi Road, Xi’an, 710072 China; 2 Research & Development Institute of Northwestern Polytechnical University in Shenzhen, Shenzhen, 518100 China; 3 Shenzhen Institutes of Advanced Electronic Materials, Shenzhen, 518100 China; 4 Key Laboratory of Flexible Electronics & Institute of Advanced Materials, Nanjing Tech University, 30 South Puzhu Road, Nanjing, 211816 China

**Keywords:** Optoelectronic devices and components, Polymers

## Abstract

Organic mechanoluminescence materials, featuring dual emission and ultralong phosphorescence characteristics, exhibit significant potential for applications in real-time stress sensing, pressure-sensitive lighting, advanced security marking techniques, and material breakage monitoring. However, due to immature molecular design strategies and unclear luminescence mechanisms, these materials remain rarely reported. In this study, we propose a valuable molecular design strategy to achieve dual-channel mechano-phosphorescence. By introducing the arylphosphine oxide group into a highly twisted molecular framework, enhanced intra- and intermolecular interactions could be achieved within rigid structures, leading to dual-channel mechanoluminescence with greatly promoted ultralong phosphorescence. Further investigations reveal the substantial boosting effect of intra- and intermolecular interactions on mechanoluminescence and ultralong phosphorescence properties by locking the highly twisted molecular skeleton. This work provides a concise and guiding route to develop novel smart responsive luminescence materials for widespread applications in material science.

## Abstract (web summary)

Dual-channel mechano-phosphorescence, characterized by dual emission and ultralong organic phosphorescence, was achieved by strengthening the intra- and intermolecular interactions in the highly twisted structures to realize a combined locking effect.

## Graphical abstract

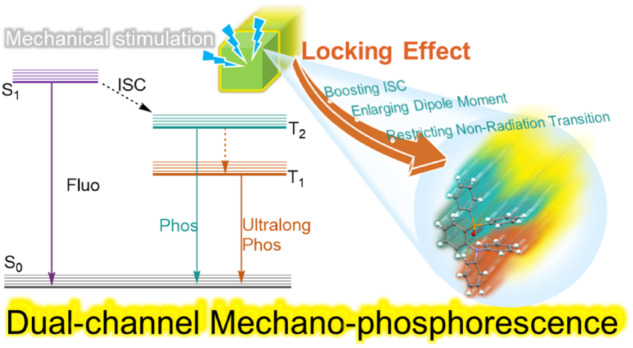

## Introduction

Mechanoluminescence (ML) materials have aroused abundant attention for their great potential applications in stress sensors, pressure-sensitive lighting, advanced security marking techniques, and material breakage monitoring^1–8^. Different from classical photoluminescence (photo-irradiation) and electroluminescence (electro-excitation), ML is ascribed to the light-generating process which is stimulated by mechano-stimulation such as pressing, grinding, and shearing^1^. Nowadays, most of the applicable ML materials are based on inorganic nanoparticles or organic/inorganic hybrid materials which exhibit strong piezoelectric effects^9–12^. Some organic light-emitting systems also feature notable ML properties in their crystalline states such as 9-anthracenemethanol, p-anisidine, resorcinol, methyl salicylate derivatives^13^, tetraphenylethene derivatives^14,15^, borate derivatives^16,17^, N-phenyl imide derivatives^18–20^, triphenylphosphine derivatives^21,22^, and phenothiazine derivatives^23,24^. The mechanism of organic ML is generally regarded as the spatial charge separation induced by piezoelectricity and then recombination to emit. Such excited-state processes require a large dipole moment, regular molecular arrangement, and strong intermolecular interactions of organic molecules. With great advances in emission color tuning, flexible device fabricating, and real-time stress sensing, organic ML materials have developed rapidly in recent years. Most of the reported ML molecules were constructed with donor-acceptor (D-A) structures to enhance the monomolecular dipole moment^2^. However, almost all organic ML materials featured fluorescence emission bands with short emission lifetimes (in nanosecond range). On the contrary, mechano-phosphorescence is more attractive in stress sensing and material breakage monitoring for its long-lasting emission features. With long-lasting ML lifetimes, the detection windows could be extended and background interference could be reduced during the stress sensing and material breakage monitoring processes.

Ultralong organic phosphorescence (UOP) materials with ultralong lifetimes of over 0.1 s are rapidly developed by a series of molecular designing strategies such as forming H-aggregation^25,26^, strengthening intermolecular interactions (C − H···π interactions, hydrogen bonding, and halogen bonding)^27–29^, boosting the degree of crystallinity^30,31^, enhancing intermolecular electronic coupling^32^, manipulation of the steric hindrance^29^, and constructing organic host-guest systems^33^. In contrast to the conventional single-channel UOPs, dual-channel UOP materials with dual emission centers exhibit real-time changing afterglow, showing unique potential applications in areas of time-dependent information displaying, high-density data storage, information encryption, and bio-imaging. These types of molecules are usually based on twisted structures and robust intermolecular interactions to restrict non-radiative transition and manipulate the molecular packing patterns^34–37^. Up until now, almost all reported UOP materials were merely excited by optical means, only rare mechano-phosphorescence examples were reported^18,38^. The molecular design strategies for mechano-phosphorescence materials are still unclear. Furthermore, as far as we know, no dual-channel mechano-phosphorescence material has yet been reported. Developing rational molecular design strategies to achieve dual-channel mechano-phosphorescence materials has attracted intense attention in both the scientific and industrial fields.

Utilizing a significantly twisted D-A molecular skeleton along with robust intra- and intermolecular interactions can be endorsed as a valid and guided approach for realizing dual-channel mechano-phosphorescence. Construction of a D-A structure can be employed to ensure an increased dipole moment of the target molecule^2,20^. Simultaneously, the highly twisted molecular skeleton can intensively inhibit molecular motions and greatly improve emission efficiency. It can also protect triplet excitons from being quenched by non-radiative transition processes attributed to some internal or external factors. Additionally, strong intra- and intermolecular interactions could further efficiently resist the non-radiative decay process and help to achieve a regular molecular arrangement that promotes ML and phosphorescence properties. As a result of the highly twisted structure and strong intra- and intermolecular interactions, a remarkable locking effect could be achieved on the target molecule. Thus, two or multiple relatively independent decay pathways may be formed to break through Kasha’s rule and contribute to dual-channel luminescence.

**Fig. 1:**
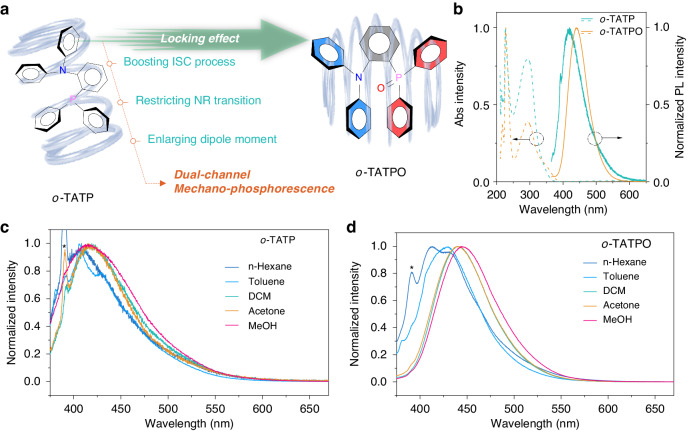
**Molecular design and photophysical properties of** ***o*-TATP and** ***o*-TATPO. a** Molecular design strategy (ISC: intersystem crossing, NR non-radiation). **b** Normalized absorption and emission spectra in DCM solutions (5 × 10^−5^ mol·L^−1^). Normalized PL spectra of *o*-TATP (**c**) and *o*-TATPO (**d**) in different solutions (5 × 10^−5^ mol·L^−1^). *The sharp peaks located between 385 and 395 nm correspond to Raman peaks of the solvents

Herein, an arylphosphine oxide-containing compound with highly twisted structure, named *o*-TATPO, have been designed and synthesized (Fig. 1a). The triphenylphosphine oxide (TPO) group was employed as an acceptor due to the remarkable electron-withdrawing ability, as well as the ability to effectively enhance the molecular dipole moment and form interactions with the donor^10,39–43^. It is revealed that both intra- and intermolecular interactions were enhanced by simply switching the triphenylphosphine (TP) group to the TPO group. Consequently, the ML properties were activated and UOP properties were greatly promoted after locking the molecules with stronger intra- and intermolecular interactions. Further photophysical studies for both fluorescence and UOP are carefully presented to explore the dual-channel phosphorescence properties. Additionally, single-crystal analyses and theoretical simulations of *o*-TATP and *o*-TATPO were carefully executed to confirm and illustrate the rational molecular design strategy and mechanism for dual-channel mechano-phosphorescence materials. This work provides a convenient and guiding strategy to design novel smart responsive luminescence materials that are expected to attract interest in the academic and industrial fields.

## Results

### Dual channel phosphorescence achieved by locking effect

In the UV-vis absorption spectra, compounds *o*-TATP and *o*-TATPO show broad absorption bands from 250 nm to 400 nm in dilute dichloromethane solution (Fig. 1b). The absorption band with the maximum at 294 nm of *o*-TATP was ascribed to the π···π^*^ transition, while that of *o*-TATPO was assignable as the π···π^*^ transition (250 ~ 320 nm) mixed with intramolecular charge transfer (ICT) (320 ~ 400 nm) from the diphenylamine moiety to the TPO moiety according to previous literature with similar structures^44^. Thus, *o*-TATPO exhibited an ICT emission compared with the locally excited (LE) one of *o*-TATP. In hexane solution, both compounds show structural emission bands. By increasing the polarities of solvents, *o*-TATP showed changeless LE emission, while *o*-TATPO displayed gradually red-shift emission from 410 to 445 nm (see Fig. 1c, d). The bathochromic luminescence induced by increased solvent polarities further demonstrated the ICT excited-state character of *o*-TATPO, endowed with a large molecular dipole moment. As listed in Table S2, the dipole moment of *o*-TATPO in monomer (4.52 Debye) exhibited a nearly twofold increase compared with that of *o*-TATP (2.46 Debye), thereby facilitating the activation of the ML property.

**Fig. 2:**
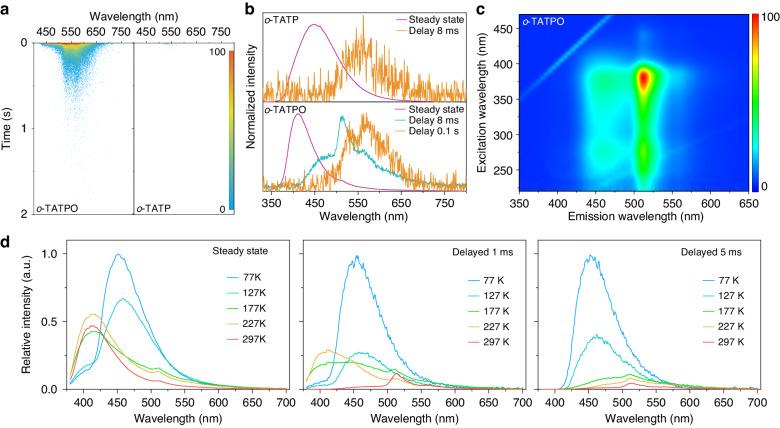
**UOP properties for the crystalline powders under 365** **nm excitation. a** Time-resolved phosphorescence spectra of *o*-TATP and *o*-TATPO. **b** Steady-state and delayed emission spectra of *o*-TATPO and *o*-TATP. **c** Excitation/delayed emission mapping of *o*-TATPO. **d** Variable-temperature steady-state and delayed spectra (delayed 1 ms and 5 ms) of *o*-TATPO

The phosphorescence lifetime and intensity experienced significant prolongation and enhancement, respectively, through the oxidation of the triphenylphosphine (TP) moiety to the TPO moiety (Fig. 2a, S1, and S2). In the crystalline state, *o*-TATP and *o*-TATPO showed blue emission bands at *ca*. 448 and 410 nm upon 365 nm excitation (Fig. 2b). After ceasing the excitation light, *o*-TATP exhibited a faint phosphorescence characterized by an emission maximum at 558 nm and a short lifetime of 20.9 ms (Fig. S3). However, a bright and ultralong greenish-yellow afterglow was attained in *o*-TATPO through oxidation. Remarkably, dual-channel phosphorescence emission bands, with maxima of 465 nm (23.24 ms) and 565 nm (191.0 ms), were observed in the delayed emission spectrum of *o*-TATPO (Figs. S3 and S4). Delayed emission mapping with various excitation wavelengths was performed to further confirm the distinct phosphorescence characteristics of these two counterparts. In contrast to the single phosphorescence band observed in *o*-TATP, *o*-TATPO consistently exhibited dual-emitting phosphorescent bands (refer to Fig. 2c and S5). Furthermore, it demonstrated distinct variations in their corresponding excitation spectra with varying excitation wavelengths (Fig. S6). This further indicates the presence of two separate phosphorescent components contributing to these dual emission bands. Owing to the disparate decay lifetimes of the two phosphorescent components, dynamic afterglows transitioning from greenish-yellow to yellow were successfully achieved in real-time, which was clearly observed in the time-resolved phosphorescence spectra (Fig. S7). To elucidate the origins of the two phosphorescent components, delayed emission spectra were obtained for the two constituent building blocks of o-TATPO (triphenylamine (TA) group and TPO group (see Figs. S8 and S9)). Each phosphorescent component of *o*-TATPO exhibited a close correspondence with the phosphorescence observed from the respective building blocks, both in vibrational structure and emission wavelength. Consequently, the dual-channel phosphorescence spectra of *o*-TATPO can be attributed to the localized phosphorescence originating from the TA and TPO moieties, respectively. Conversely, in *o*-TATP, only TA moiety contributed to the phosphorescence process (Fig. S10). The inefficient internal conversion process between the two phosphorescence decay channels can be ascribed to the locking effect induced by the highly twisted structure and robust intra- and intermolecular interactions among the building blocks, according to previous literature^29^.

To further investigate the dual-channel phosphorescence properties, studies on variable-temperature steady-state and delayed spectra were conducted for *o*-TATPO and *o*-TATP in both crystalline state and doped polymethyl methacrylate (PMMA) films. *o*-TATPO displayed multiple emission featuring both fluorescent and phosphorescent components in the steady-state and delayed spectra (delayed by 1 ms) at varying temperatures (Fig. 2d). As a CT type molecule with highly twisted structure, *o*-TATPO exhibited distinct delayed fluorescence at 410 nm, demonstrating a thermally activated characteristic. To elucidate the phosphorescent property of *o*-TATPO clearly, variable-temperature spectra delayed by 5 ms were performed to eliminate the delayed fluorescence. Remarkably, lower temperatures exhibited a more pronounced enhancing effect on the phosphorescence, peaking at 465 nm and originating from the TPO moiety, as compared with the lower-energy phosphorescent component from the TA moiety. The restricted thermal motion of *o*-TATPO at lower temperatures (<127 K) effectively blocked the nonradiative transition pathway, leading to the dominance of phosphorescence from the TPO moiety in the delayed luminescence. In the case of *o*-TATP, a distinctive phosphorescent peak also emerged in the higher energy region at low temperatures due to the obstructed nonradiative transition pathway (Fig. S11). However, in contrast to *o*-TATPO, *o*-TATP only demonstrated single-channel phosphorescence at room temperature. It was revealed that the highly twisted structure and robust intra- and intermolecular interactions played a crucial role in the formation of dual-channel phosphorescence. The phosphorescent properties of *o*-TATPO were also investigated in degassed toluene solutions and PMMA neat films to elucidate how the dual-channel phosphorescence changes with a reduction in the strength of intermolecular interactions. As depicted in Fig. S13, ultralong phosphorescence is absent in the PMMA films of *o*-TATPO. This absence suggests that the ultralong lifetime is quenched when intermolecular interactions weaken during the transition from a crystalline environment to a PMMA matrix. The reduction in strength of these interactions in the PMMA matrix hinders the generation of ultralong phosphorescence. At 77 K, *o*-TATPO consistently exhibited single-channel phosphorescence originating from the TPO moiety in both solution and neat film states, rather than the dual-channel phosphorescence observed in the crystalline state (Figs. S14–S16). These findings underscore the crucial role played by the enhanced intra- and intermolecular interactions resulting from the introduction of the P = O polar bond during crystallization in the formation of dual-channel ultralong phosphorescence.

### Dual-channel mechano-phosphorescence of *o*-TATPO

**Fig. 3:**
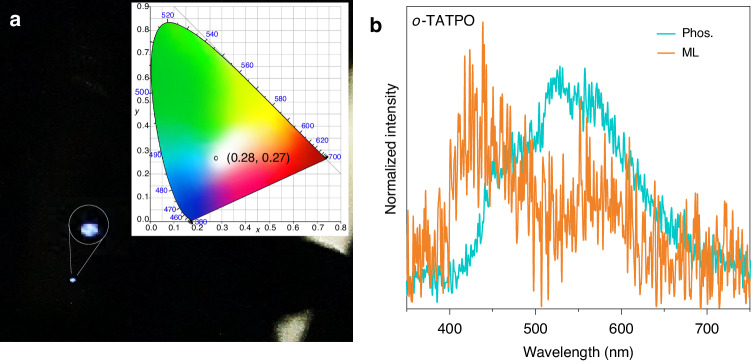
**Dual-channel ML properties of crystalline** ***o*-TATPO. a** ML Photograph taken in a dark condition and the corresponding CIE coordinate. **b** Phosphorescence and ML spectra of o-TATPO at room temperature

In a further set of experiments, ML studies for compounds *o*-TATP and *o*-TATPO were carried out on their crystalline powers. Expectedly, no ML phenomenon was observed for compound *o*-TATP, while *o*-TATPO showed a distinct ML phenomenon (Fig. 3). The activation of ML could be attributed to the greatly enhanced dipole moment and the rigid molecular structure of *o*-TATPO. Notably, crystalline *o*-TATPO displayed distinct bluish-white emission with a CIE coordinate of (0.28, 0.27) under mechanical stimulation (Fig. 3a). Most reported ML materials typically manifest a single emission peak for fluorescence or phosphorescence. However, *o*-TATPO demonstrated dual-channel ML with emission maxima at 450 nm and 565 nm. As illustrated in Fig. 3b, the dual emission in ML stemmed from the dual-channel phosphorescence, though with a different emission color compared with that of the UOP due to the presence of a small proportion of fluorescent components. For all we know, dual-channel mechano-phosphorescence has not been reported before. In addition, the dual-channel mechano-phosphorescence could be ascribed to efficient ISC processes and the rigid environment of excited states. To illustrate the origin of dual-channel mechano-phosphorescence, single crystal analyses and theoretical simulation studies of these two compounds were performed as follows.

### Single crystal analysis for dual-channel mechano-phosphorescence

**Fig. 4:**
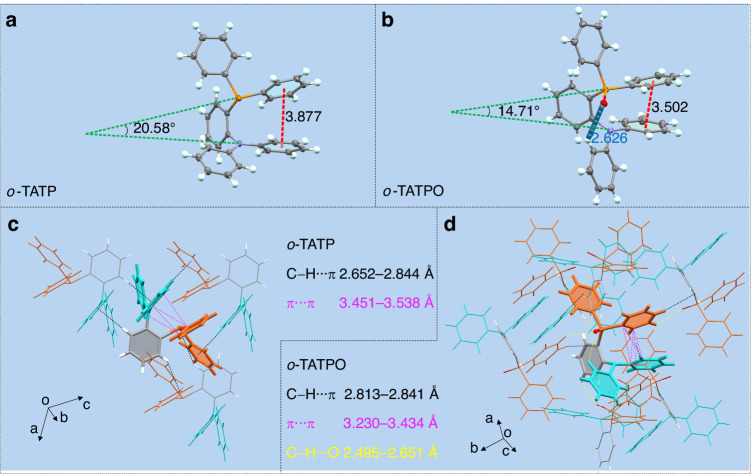
**Single crystal structures and interactions of** ***o*-TATP and** ***o*-TATPO.** Single crystal structures of *o*-TATP (**a**) and *o*-TATPO (**b**). **c**, **d** Intra- and intermolecular interactions with distances short of 3.60 Å

Single crystals of *o*-TATP and *o*-TATPO were obtained by liquid phase diffusion method with dichloromethane-hexane and dichloromethane-methanol mixed solvent systems respectively. Details of single crystal analysis data are listed in Tables S3−S8. Figure 4 illustrates twisted conformations and contrasting intra- and intermolecular interactions of *o*-TATP and *o*-TATPO. Within *o*-TATP, a relatively longer distance of 3.88 Å was measured between the two adjacent phenyl rings of the TA moiety and diphenylphosphine moiety (Fig. 4a). In the case of *o*-TATPO, a robust C − H···O intramolecular hydrogen bond (2.63 Å) was formed, contributing to a highly twisted structure and bringing the two adjacent phenyl rings into close proximity (with a shorter distance of 3.50 Å) (see Fig. 4b). Simultaneously, the dihedral angle between the labeled face-to-face phenyl rings was decreased from 20.58^o^ (for *o*-TATP) to 14.71^o^ (for *o*-TATPO). As a result, stronger intramolecular π···π interactions were generated in *o*-TATPO based on these prerequisites, compared with that of *o*-TATP, as shown in Fig. 4c, d. Hence, the substitution of the TP group with the TPO group led to a significant intensification of intramolecular interactions, and then induced a highly twisted structure in *o*-TATPO. Besides the highly twisted structure and robust intramolecular interactions, the pronounced intermolecular interactions among adjacent molecules demonstrated a significant enhancing effect on the development of dual-channel mechano-phosphorescence. This allowed for the regulation of molecular arrangement and resistance against non-radiative decay processes. As depicted in Fig. 4c, o-TATP exhibited fragile connections with only three neighboring molecules in two dimensions, forming several C − H···π interactions with distances ranging from 2.65 to 2.84 Å. In contrast, *o*-TATPO exhibited a strong binding affinity with the surrounding molecules in the spatial domain. Due to the introduction of the P = O polar bond, it was constrained by four robust C − H···O intermolecular hydrogen bonds (with distances ranging from 2.49 to 2.65 Å) and four C − H···π interactions (with distances ranging from 2.81 to 2.84 Å) formed with neighboring six molecules (refer to Fig. 4d). Based on the quantity and strength of the intra- and intermolecular interactions discussed above, *o*-TATPO exhibited a more tightly packing structure compared with *o*-TATP (refer to Figs. S17 and S18). As a result, the combined effects of these strong interactions and the highly twisted structure in *o*-TATPO contributed to a notable locking effect, effectively suppressing non-radiative decay pathways such as molecular rotations and vibrations. This substantial restriction allowed triplet excitons to decay along separate paths, thereby leading to a significantly enhanced dual-channel phosphorescence. Moreover, heightened intra- and intermolecular interactions in *o*-TATPO may facilitate the accumulation of dipole moments as molecules aggregate, thereby activating the ML property in the crystalline state.

### Theoretical simulations and proposed mechanism for dual-channel mechano-phosphorescence

**Fig. 5:**
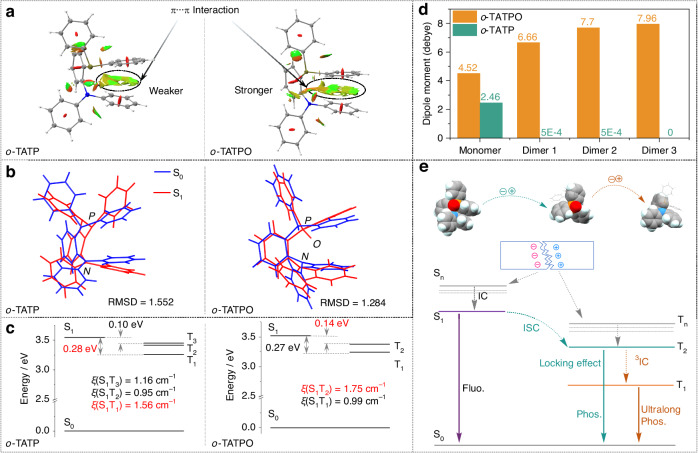
**Theoretical simulation results and proposed dual-channel mechano-phosphorescence mechanism. a** RDG isosurface maps of *o*-TATP and *o*-TATPO in monomers with an isovalue of 0.5. **b** Root mean square displacement/deviation (RMSD) of *o*-TATP and *o*-TATPO. **c** Spin-orbital coupling (SOC) matrix elements (ξ) of *o*-TATP and *o*-TATPO. **d** Dipole moment calculated in monomer and dimer states based on single crystal structures. **e** Jablonski diagram for proposed photophysical processes of the dual-channel mechano-phosphorescence in *o*-TATPO

To gain more detailed and insightful information about the impact of the locking effect on dual-channel mechano-phosphorescence, we conducted theoretical simulations for these two compounds. The simulations included non-covalent interactions, spin-orbit coupling (SOC) matrix, and natural transition orbitals (NTO) analyses, all based on their crystal structures. Figure 5a shows the reduced density gradient (RDG) isosurface maps with an isovalue of 0.5 for the monomers. In the visualized analyses of intramolecular non-covalent interactions in single molecular states, π···π intramolecular interaction region in *o*-TATPO was larger than that in *o*-TATP, indicating stronger intramolecular π···π interactions in the locked molecules. To further ascertain the locking effect in the excited processes, the conformational changes between the ground states (S_0_) and excited states (S_1_) were depicted in Fig. 5b. The root mean square displacement/deviation (RMSD) values of these two counterparts were also calculated. For *o*-TATP, the conformational distortion during the excited processes predominantly occurred in the TP moiety, indicating that excitons would decay from the TA moiety with less conformational distortion and emit light. After molecular oxidation, the overall conformational distortion is minimized, resulting in a decreased RMSD value from 1.552 to 1.284. The reduced value indicates fewer vibrations of the excited states, resulting in a less efficient non-radiative decay in *o*-TATPO, attributed to the strong locking effect. The highly twisted conformation of the emitters facilitated the ISC process while concurrently diminishing non-radiative decay. This suggests the existence of multiple radiative transition pathways for excitons in *o*-TATPO. Additionally, the SOC matrixes of *o*-TATP and *o*-TATPO were investigated, as depicted in Fig. 5c. The large SOC matrix elements can be ascribed to the high ratio of heteroatoms (N, P, and O) in these compounds, owing to the presence of n orbitals. Moreover, NTO analyses of these two compounds were conducted to assess the features of their excited states. As depicted in Figs. S19 and S20, the electron orbitals of *o*-TATP and *o*-TATPO displayed a hybrid nature of n and π orbitals, attributable to the twisted conformations of the two molecules. The hybrid orbitals could promote the ISC process with large SOC, in accordance with the El-Sayed rules. In the case of *o*-TATP, the largest SOC matrix element was S_1_-T_1_ (∆*E*_ST_ = 0.28 eV). Following oxidation to *o*-TATPO, the largest SOC matrix element changed to S_1_-T_2_ (∆*E*_ST_ = 0.14 eV). The narrow singlet-triplet energy gap for *o*-TATPO could further facilitate the formation of triplet excitons, leading to efficient phosphorescence. Significantly, theoretical simulation results of IGM isosurface maps in dimers of these two counterparts also revealed a contrast in the intermolecular interactions (see Figs. S21 and S22). In comparison with *o*-TATP, additional C − H···O intermolecular hydrogen bonds were identified in dimers of *o*-TATPO, in addition to the common C − H···π interactions, which further indicated the strengthening of intermolecular interactions after oxidation. Thus, all the aforementioned findings suggest that the effective locking effect, attributed to the highly twisted structure and strong interactions, exerts a significant enhancing influence on the phosphorescence of *o*-TATPO. This enhancement was reflected in the boosted phosphorescence quantum yield, prolonged decay lifetimes, and the achievement of dual-channel phosphorescence. Furthermore, the packing mode of the molecules was changed from a centrosymmetric space group (*P*-1, *o*-TATP) to a non-centrosymmetric one (*P*bca, *o*-TATPO), attributable to the reinforced interactions caused by oxidation. As illustrated in Fig. 5d, leveraging the distinctive packing mode, the dipole moments of *o*-TATPO in dimers were significantly amplified. This accumulation in the crystalline state could, therefore, reach a certain threshold to activate the ML property.

Particularly, we note that the ML spectrum of *o*-TATPO was mainly composed of phosphorescence. It could be attributed to the charge separation and recombination processes caused by the piezoelectric effect under mechano-excited mode, leading to the generation of a higher proportion of triplet excitons. Meanwhile, part of singlet excitons was also converted into triplet excitons through the ISC process. Consequently, *o*-TATPO exhibited a higher propensity to generate phosphorescence under mechano-excited mode. Such an example has also been reported in previous literature^16^. In addition, as distinct from the Phosphorescence (PL) spectrum, the short-lived phosphorescent component was stronger than the long-lived one in the ML spectrum. It can be inferred that intermolecular interactions on the fractured surface of the crystal were disrupted, thereby compromising the rigid microenvironment of the molecules to a certain extent. This disruption, in turn, enhanced the non-radiative transitions of triplet excitons. It was noted that the short-lived phosphorescent component in *o*-TATPO was predominantly constrained by a highly twisted structure and robust intramolecular interactions. As a result, the disruption of intermolecular interactions and the breakdown of the rigid microenvironment were more likely to diminish the long-lived phosphorescent component. This led to the unequal ratios of the two phosphorescent components between the PL and ML spectra.

To elucidate the locking effect strategy and obtain a comprehensive understanding of the mechanism underlying dual-channel mechano-phosphorescence, the Jablonski diagram illustrating the proposed photophysical processes for o-TATPO is summarized in Fig. 5e. The efficient ISC process was promoted by the large SOC, attributed to a high-ratio heteroatom effect to n orbitals for harvesting triplet excitons. The robust locking effect minimized the vibrations of the excited states, and then resulted in a less efficient non-radiative transition. Consequently, certain triplet excitons chose direct decay from a higher excited state (TPO moiety), emitting fast phosphorescence. The majority, however, underwent a decelerated internal transition process, relaxing to the lowest triplet excited state (TA moiety), thereby resulting in ultralong phosphorescence. As a combined outcome, with the highly twisted conformation and rigid environment in the *o*-TATPO, multiple relatively independent decay pathways could be formed to break through Kasha’s rule and contribute to dual-channel mechano-phosphorescence.

## Discussion

In conclusion, dual-channel mechano-phosphorescence was successfully achieved through the reinforcement of intra- and intermolecular interactions, coupled with the establishment of a locking effect within a highly twisted framework. Photophysical studies revealed the distinctive dual-channel mechano-phosphorescence arising from the triplet localized excited states of TA and TPO moieties, respectively. Single-crystal analyses and theoretical simulations provided insights into the locking effect of intra- and intermolecular interactions, as well as fast ISC process. This study presents a rare example of a dual-channel mechano-phosphorescent material and proposes a suggestive molecular design strategy. The dual-channel mechano-phosphorescent material is anticipated to attract significant interest in the fields of real-time stress sensing, time-dependent information display, pressure-sensitive lighting, advanced security marking techniques, and material breakage monitoring.

## Materials and methods

General Methods, Materials and Syntheses are shown in the Supplementary Information. The accession numbers for the crystallographic data reported in this paper are CCDC: 2061348 and 2061349. These data can be obtained free of charge from the Cambridge Crystallographic Data Center at https://www.ccdc.cam.ac.uk/structures.

### Supplementary information

Supporting information

CIF file for o-TATP

CIF file for o-TATPO

checkCIF_o-TATP

checkCIF_o-TATPO

## References

[1] Xie YJ, Li Z (2018). Triboluminescence: recalling interest and new aspects. Chem.

[2] Mukherjee S, Thilagar P (2019). Renaissance of organic triboluminescent materials. Angew. Chem. Int. Ed..

[3] Camara CG (2008). Correlation between nanosecond X-ray flashes and stick–slip friction in peeling tape. Nature.

[4] Eddingsaas NC, Suslick KS (2006). Light from sonication of crystal slurries. Nature.

[5] Huang ZF (2023). Smart mechanoluminescent phosphors: a review of strontium-aluminate-based materials, properties, and their advanced application technologies. Adv. Sci..

[6] Zhuang YX, Xie RJ (2021). Mechanoluminescence rebrightening the prospects of stress sensing: a review. Adv. Mater..

[7] Xie ZL (2023). Realizing photoswitchable mechanoluminescence in organic crystals based on photochromism. Adv. Mater..

[8] Hou B (2022). An interactive mouthguard based on mechanoluminescence-powered optical fibre sensors for bite-controlled device operation. Nat. Electron..

[9] Jeong SM (2014). Bright, wind-driven white mechanoluminescence from zinc sulphide microparticles embedded in a polydimethylsiloxane elastomer. Energy Environ. Sci..

[10] Hirai Y (2017). Triboluminescence of lanthanide coordination polymers with face-to-face arranged substituents. Angew. Chem. Int. Ed..

[11] Qian X (2018). Printable skin-driven mechanoluminescence devices via nanodoped matrix modification. Adv. Mater..

[12] Peng D (2020). A ZnS/CaZnOS heterojunction for efficient mechanical-to-optical energy conversion by conduction band offset. Adv. Mater..

[13] Hardy GE (1977). Triboluminescence spectroscopy of aromatic compounds. J. Am. Chem. Soc..

[14] Xu BJ (2015). Very bright mechanoluminescence and remarkable mechanochromism using a tetraphenylethene derivative with aggregation-induced emission. Chem. Sci..

[15] Wang C (2016). A stable tetraphenylethene derivative: aggregation-induced emission, different crystalline polymorphs, and totally different mechanoluminescence properties. Mater. Horiz..

[16] Yang J (2017). AIEgen with fluorescence-phosphorescence dual mechanoluminescence at room temperature. Angew. Chem. Int. Ed..

[17] Sun QK (2018). Bright NUV mechanofluorescence from a terpyridine-based pure organic crystal. Chem. Commun..

[18] Li JA (2018). Transient and persistent room-temperature mechanoluminescence from a white-light-emitting AIEgen with tricolor emission switching triggered by light. Angew. Chem. Int. Ed..

[19] Nakayama H (2012). Crystal structures and triboluminescence based on trifluoromethyl and pentafluorosulfanyl substituted asymmetric *N*-phenyl imide compounds. Chem. Mater..

[20] Nishida JI (2016). Phthalimide compounds containing a trifluoromethylphenyl group and electron-donating aryl groups: color-tuning and enhancement of triboluminescence. J. Org. Chem..

[21] Hardy GE (1978). Structure and triboluminescence of polymorphs of hexaphenylcarbodiphosphorane. J. Am. Chem. Soc..

[22] Xie ZL (2018). Weak interactions but potent effect: tunable mechanoluminescence by adjusting intermolecular C-H⋯π interactions. Chem. Sci..

[23] Yang J (2017). Elucidating the excited state of mechanoluminescence in organic luminogens with room-temperature phosphorescence. Angew. Chem. Int. Ed..

[24] Neena KK (2017). Diarylboryl-phenothiazine based multifunctional molecular siblings. Chem. Commun..

[25] Lucenti E (2017). H-aggregates granting crystallization-induced emissive behavior and ultralong phosphorescence from a pure organic molecule. J. Phys. Chem. Lett..

[26] An ZF (2015). Stabilizing triplet excited states for ultralong organic phosphorescence. Nat. Mater..

[27] He ZK (2017). White light emission from a single organic molecule with dual phosphorescence at room temperature. Nat. Commun..

[28] Yang Z (2020). Boosting the quantum efficiency of ultralong organic phosphorescence up to 52% via intramolecular halogen bonding. Angew. Chem. Int. Ed..

[29] Zhang ZY, Liu Y (2019). Ultralong room-temperature phosphorescence of a solid-state supramolecule between phenylmethylpyridinium and cucurbit[6]uril. Chem. Sci..

[30] Gong YY (2015). Achieving persistent room temperature phosphorescence and remarkable mechanochromism from pure organic luminogens. Adv. Mater..

[31] Bolton O (2011). Activating efficient phosphorescence from purely organic materials by crystal design. Nat. Chem..

[32] Yang ZY (2016). Intermolecular electronic coupling of organic units for efficient persistent room-temperature phosphorescence. Angew. Chem. Int. Ed..

[33] Kabe R, Adachi C (2017). Organic long persistent luminescence. Nature.

[34] Chen JR (2019). Achieving dual-emissive and time-dependent evolutive organic afterglow by bridging molecules with weak intermolecular hydrogen bonding. Adv. Opt. Mater..

[35] Ling K (2019). Controllable multiemission with ultralong organic phosphorescence in crystal by isomerization. Adv. Opt. Mater..

[36] Gu L (2018). Dynamic ultralong organic phosphorescence by photoactivation. Angew. Chem. Int. Ed..

[37] Liu Y (2021). Robust white-light emitting and multi-responsive luminescence of a dual-mode phosphorescence molecule. Adv. Opt. Mater..

[38] Zhang XP (2019). Ultralong UV/mechano-excited room temperature phosphorescence from purely organic cluster excitons. Nat. Commun..

[39] Biju S (2013). Brilliant photoluminescence and triboluminescence from ternary complexes of Dy^III^ and Tb^III^ with 3-phenyl-4-propanoyl-5-isoxazolonate and a bidentate phosphine oxide coligand. Inorg. Chem..

[40] Chandra BP, Zink JI (1982). Triboluminescence of crystals containing the triphenyl group. J. Phys. Chem..

[41] Yang XL, Zhou GJ, Wong WY (2015). Functionalization of phosphorescent emitters and their host materials by main-group elements for phosphorescent organic light-emitting devices. Chem. Soc. Rev..

[42] Zhou GJ (2008). Manipulating charge-transfer character with electron-withdrawing main-group moieties for the color tuning of iridium electrophosphors. Adv. Funct. Mater..

[43] Zhou GJ (2010). Metallophosphors of platinum with distinct main-group elements: a versatile approach towards color tuning and white-light emission with superior efficiency/color quality/brightness trade-offs. J. Mater. Chem..

[44] Liu XY (2018). Novel *o*-D-π-A arylamine/arylphosphine oxide hybrid hosts for efficient phosphorescent organic light-emitting diodes. Org. Electron..

