# Supplementary material for: Dual-channel mechano-phosphorescence: a combined locking effect with twisted molecular structures and robust interactions
Source: Light Sci Appl. 2024 Apr 8;13:85. doi: 10.1038/s41377-024-01421-5 (PMC11001961; doi:10.1038/s41377-024-01421-5)
Supplement: Supplementary file 1 — Supporting information [file 41377_2024_1421_MOESM1_ESM.docx]

**Supplementary Information for**

**Dual-channel Mechano-phosphorescence: A Combined Locking Effect with Twisted Molecular Structures and Robust Interactions**

Zongliang Xie^1,4,5^, Zhu Mao^2,5^, Hailan Wang^1^, Yuxin Xiao^1^, Xiayu Zhang^1^, Tao Yu^1,4,^*, Zhongfu An^3,^* & Wei Huang^1,^^3,^*

^1^Frontiers Science Center for Flexible Electronics, Xi’an Institute of Flexible Electronics & Xi’an Institute of Biomedical Materials and Engineering, Northwestern Polytechnical University, 127 West Youyi Road, Xi'an 710072, China

^2^Shenzhen Institutes of Advanced Electronic Materials, Shenzhen 518100, China

^3^Key Laboratory of Flexible Electronics & Institute of Advanced Materials, Nanjing Tech University, 30 South Puzhu Road, Nanjing 211816, China

^4^Research & Development Institute of Northwestern Polytechnical University in Shenzhen, Shenzhen 518100, China

^5^These authors contributed equally.

*Correspondence: [iamtyu@nwpu.edu.cn](mailto:iamtyu@nwpu.edu.cn) (T.Y.), [iamzfan@njtech.edu.cn](mailto:iamzfan@njtech.edu.cn)(Z.A.), [vc@nwpu.edu.cn](mailto:vc@nwpu.edu.cn)(W.H.)

Table of Contents

[Table of Contents 2](#_Toc150856157)

[Experimental Procedures 3](#_Toc150856158)

[General Methods 3](#_Toc150856159)

[Materials and Syntheses 3](#_Toc150856160)

[Supplementary Information 4](#_Toc150856164)

[Supplemental Figures 4](#_Toc150856165)

[Single crystal data 15](#_Toc150856191)

[Characterization of chemical structure 18](#_Toc150856200)

[References 24](#_Toc150856213)

Experimental Procedures

General Methods

^1^H NMR, ^13^C NMR, and ^31^P NMR spectra for the materials were performed on a Bruker Avance NEO 500 Nuclear Magnetic Resonance Spectrometer with Chloroform-*d* as solvent and tetramethylsilane (TMS) as the internal standard. High-resolution Mass spectra (MS) were recorded on an Exactive GC high-resolution mass spectrometer. TG-DSC Q600 (TA Instruments) was used to obtain the differential thermogravimetry curves. PXRD data were obtained on a Bruker X-ray diffractometer (D8 Advance) by using a Cu Kα (*λ*= 0.154184 nm) X-ray source in the circumstances of 40 kV and 30 mA, with a speed of 10° (2θ) per 1 min. Single-crystal X-ray analyses were collected using a Bruker D8 Venture X-ray Single Crystal Diffractometer with a (Cu) X-ray source. UV-vis absorption spectra, photoluminescence (PL) spectra and phosphorescence spectra were obtained on a UV-vis spectrometer (Hitachi U-3900H) and a Hitachi F-7100 fluorescence spectrophotometer. Phosphorescence lifetimes were collected using an Edinburgh Instruments Spectrofluorometer (FLS 1000). Absolute PL quantum yields were measured on a Hamamatsu absolute PL quantum yield spectrometer C11347-11 Quantaurus-QY (The phosphorescence quantum yields of *o*-TATP and *o*-TATPO were calculated from the proportion in their steady-state emission spectra and phosphorescence spectra). Mechanoluminescence (ML) spectra and delayed emission spectra were measured on an Ocean Optic QE 65Pro spectrometer with Ocean Optic reflection probes R600-125F, respectively. TD-DFT calculations at B3LYP functional with 6-311G* basis set level were performed based on single crystal structure in Gaussian 09 software on the grounds of previous literatures^1,2^.

Materials and syntheses

All reagents and solvents were purchased from Aladdin, Titan, or Adamas, and were used as received. Synthetic routes for *o*-TATP and *o*-TATPO were described in Scheme S1. The intermediate compound *o*-TAI and target compounds *o*-TATP and *o*-TATPO were synthesized following the previous literatures^3,4^. All the products were purified by column chromatography and were confirmed by ^1^H NMR, ^13^C NMR, and ^31^P NMR spectra and elemental analysis.

**Scheme S1.** Synthetic routes for *o*-TATP and *o*-TATPO.

**Synthesis of 2-(diphenylphosphaneyl)-N, N-diphenylaniline (*o*-TATP)**

*o*-TAI (3.00 g, 8.08 mmol) was dissolved in anhydrous tetrahydrofuran (THF) (50 mL). Then *n*-butyl lithium (5.51 mL, 8.82 mmol, 1.6 M) was added dropwise under the N_2_ atmosphere. After stirring at -78 ^o^C for 1 h, chlorodiphenyl phosphine (1.62 g, 7.35 mmol) in 10 mL anhydrous THF was added in sequence (over 10 min) to the solution. The reaction mixture was stirred at -78 ^o^C for an additional 2 h and warmed up to room temperature gradually. After stirring overnight, 100 mL water was added to quench the reaction. The resulting mixture was extracted with dichloromethane three times. The separated organic layer was evaporated under reduced pressure to get the crude product as a light-yellow powder. Chromatography on a silica-gel column with dichloromethane-hexane (1/3, v/v) as eluent was then performed for further purification to get white powder of *o*-TATP (2.31 g, 5.38 mmol) with 73.2% yield. ^1^H NMR (500 MHz, DMSO-*d_6_*) δ 7.50 – 7.45 (m, 1H), 7.33 – 7.25 (m, 7H), 7.21 – 7.17 (m, 1H), 7.13 – 7.08 (m, 4H), 7.07 – 7.01 (m, 5H), 6.86 – 6.80 (m, 2H), 6.79 – 6.72 (m, 4H). ^13^C NMR (126 MHz, Chloroform-*d*) δ 151.57, 151.38, 148.06, 137.78, 137.66, 137.03, 136.93, 136.37, 133.82, 133.66, 130.71, 130.34, 128.75, 128.29, 128.20, 126.29, 122.55, 121.64. ^31^P NMR (202 MHz, Chloroform-*d*): δ = -16.38. High resolution EI-MS: m/z found: 428.16124 [M]^+^; calcd for C_30_H_24_NP: 429.16464.

**Synthesis of (2-(diphenylamino)phenyl) diphenylphosphine oxide (*o*-TATPO)**

To a solution of *o*-TATP (1.50 g, 3.49 mmol) in dichloromethane (DCM) (50 mL), 30% hydrogen peroxide (H_2_O_2_, 5 mL) was added dropwise. The reaction mixture was stirred and reacted overnight at room temperature. The resulting mixture was poured into 100 mL water. The organic layer was separated, dried, and then concentrated under reduced pressure. The crude product was purified by Chromatography on a silica-gel column with DCM-MeOH (30/1, v/v) as eluent to afford the target product of *o*-TATPO as a white powder (1.25 g, 2.81 mmol) with 80.3% yield. ^1^H NMR (500 MHz, DMSO-*d_6_*) δ 7.67 (t, 1H), 7.45 (t, 6H), 7.40 – 7.32 (m, 5H), 7.30 – 7.22 (m, 2H), 7.00 (t, 4H), 6.78 (t, 2H), 6.73 – 6.64 (m, 4H). ^13^C NMR (126 MHz, Chloroform-*d*) δ 151.00, 148.14, 136.14, 136.05, 133.51, 133.49, 133.18, 132.74, 132.68, 132.34, 132.16, 131.67, 131.59, 131.36, 131.15, 131.13, 128.46, 128.10, 128.00, 125.65, 125.55, 123.06, 122.01. ^31^P NMR (202 MHz, Chloroform-*d*): δ = 25.76. High resolution EI-MS: m/z found: 445.15833 [M]^+^; calcd for C_30_H_24_NPO: 445.15955.

Supporting Information

Supplementary Figures


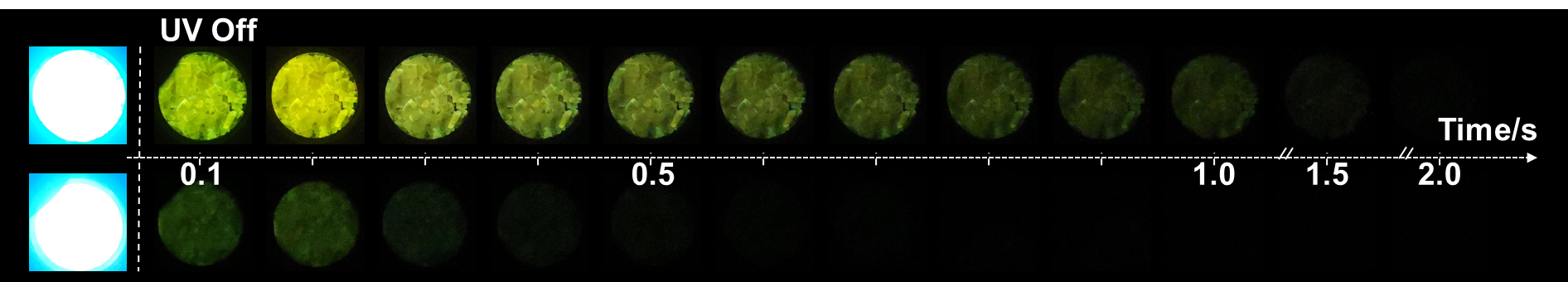


**Figure S1.** Photographs of *o*-TATPO and *o*-TATP taken under a 365 nm UV lamp on and off.


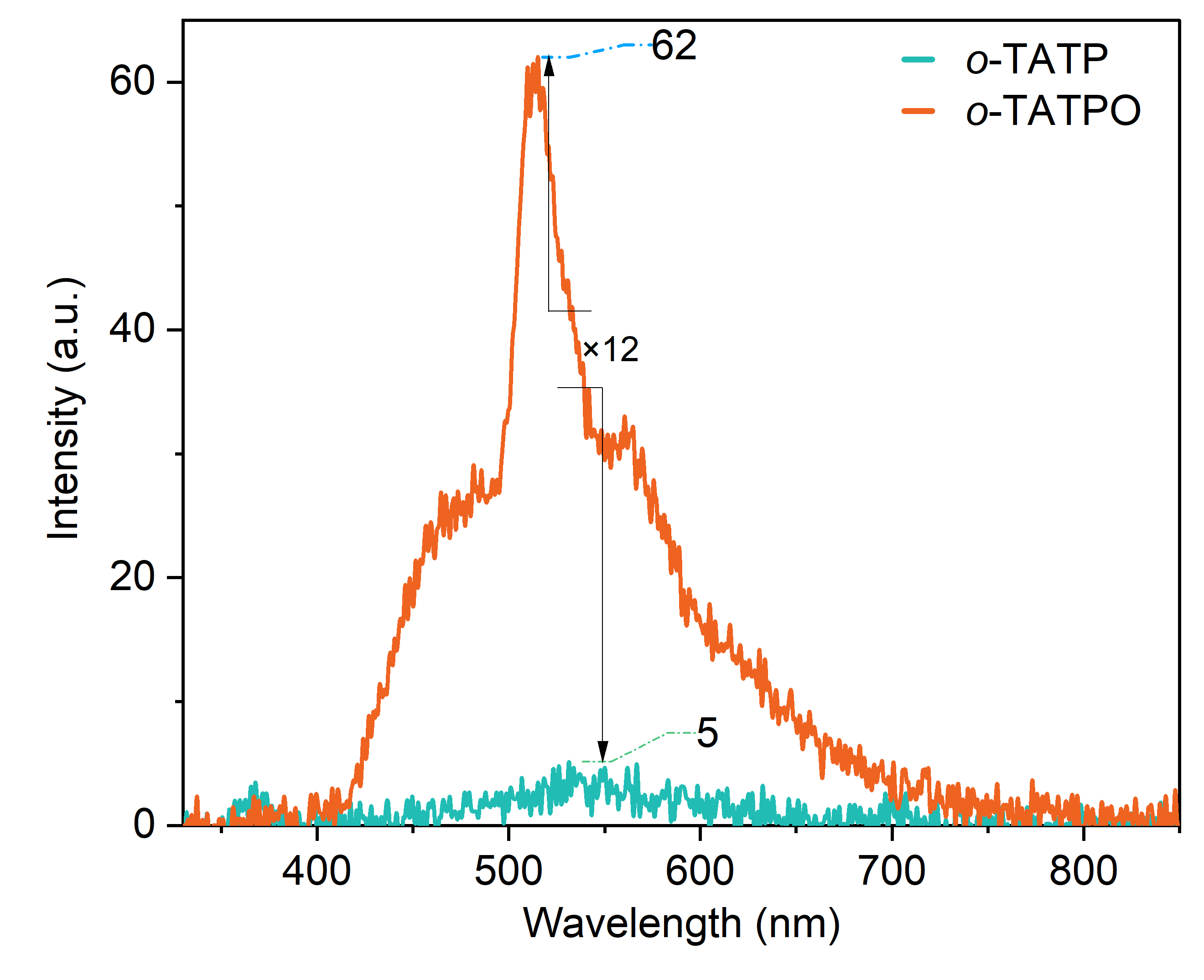


**Figure S2.** Delayed spectra at 8 ms after stoppage of 365 nm excitation.


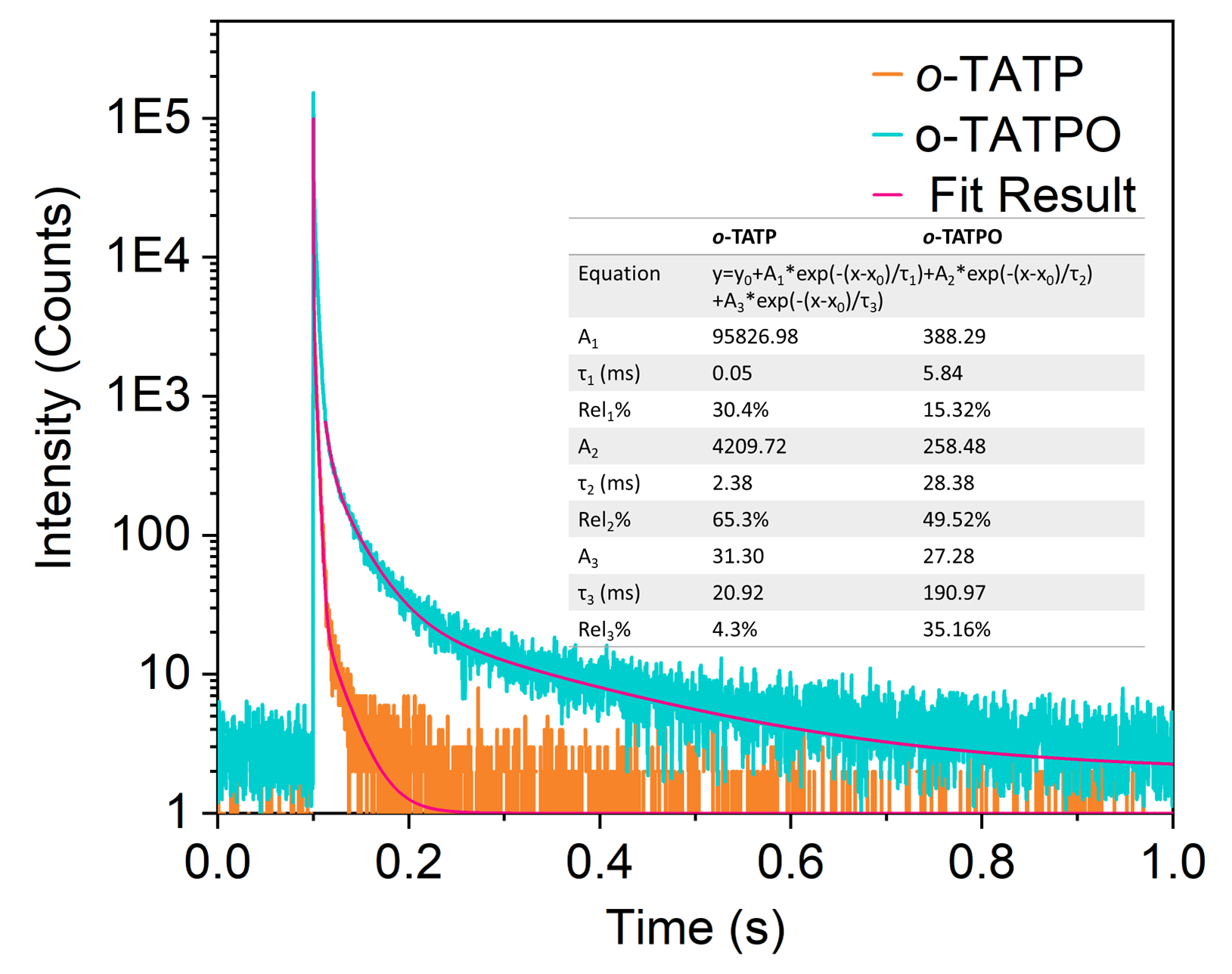


**Figure S3.** Time-resolved decay curves of *o*-TATPO and *o*-TATP


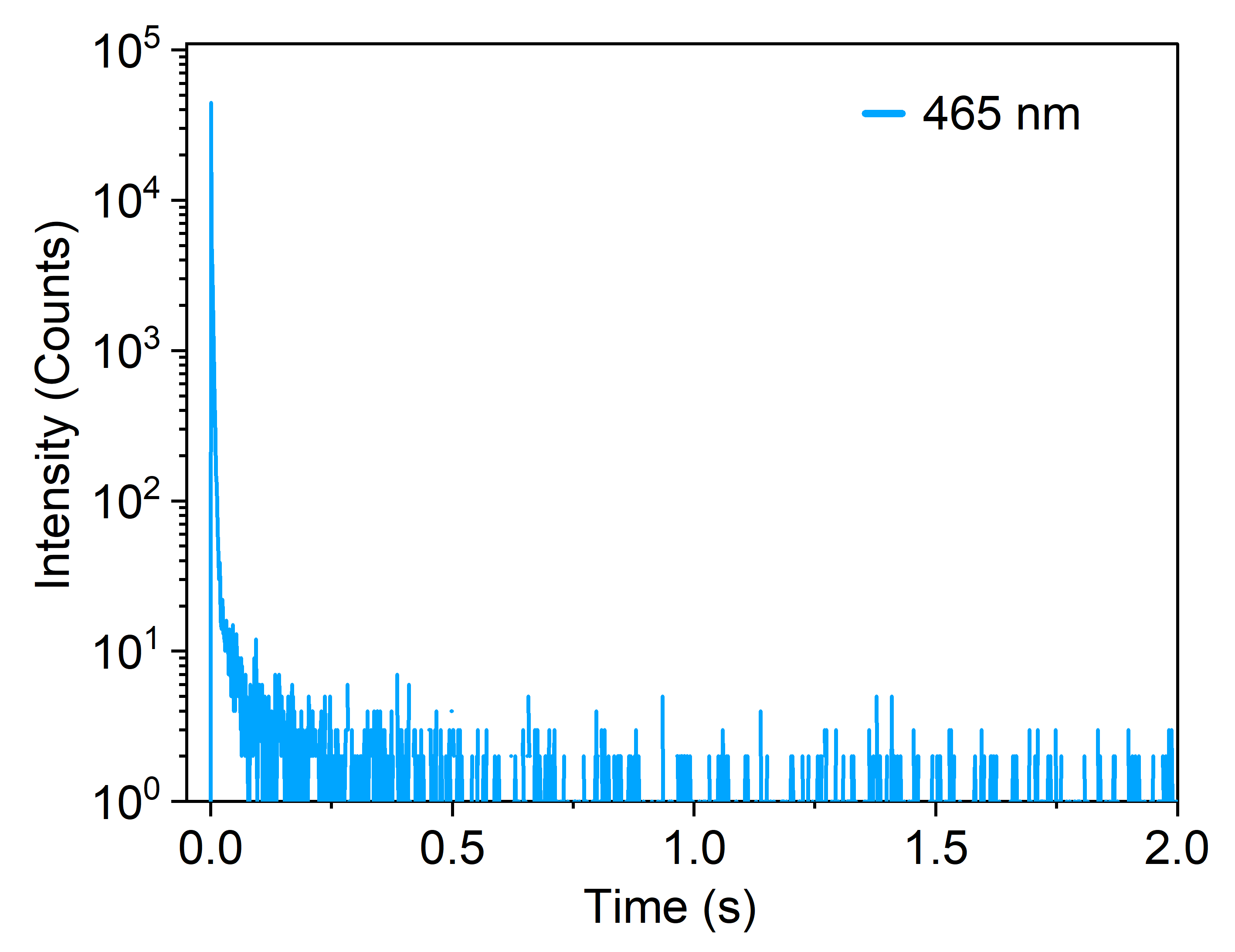


**Figure S4.** Time-resolved decay curves of *o*-TATPO at 465 nm excited at 365 nm.


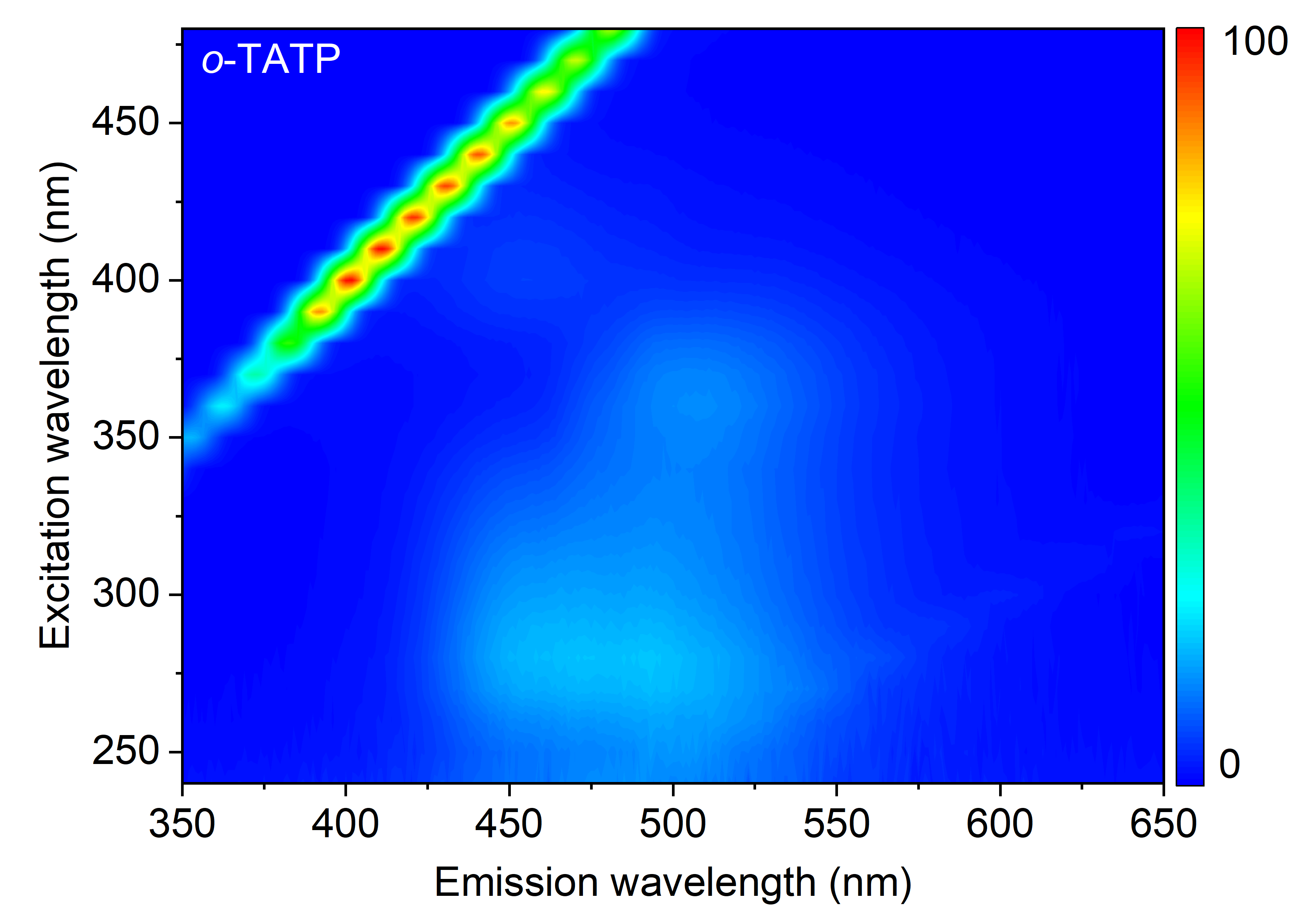


**Figure S5.** Excitation-delayed emission mapping of *o*-TATP under ambient conditions.


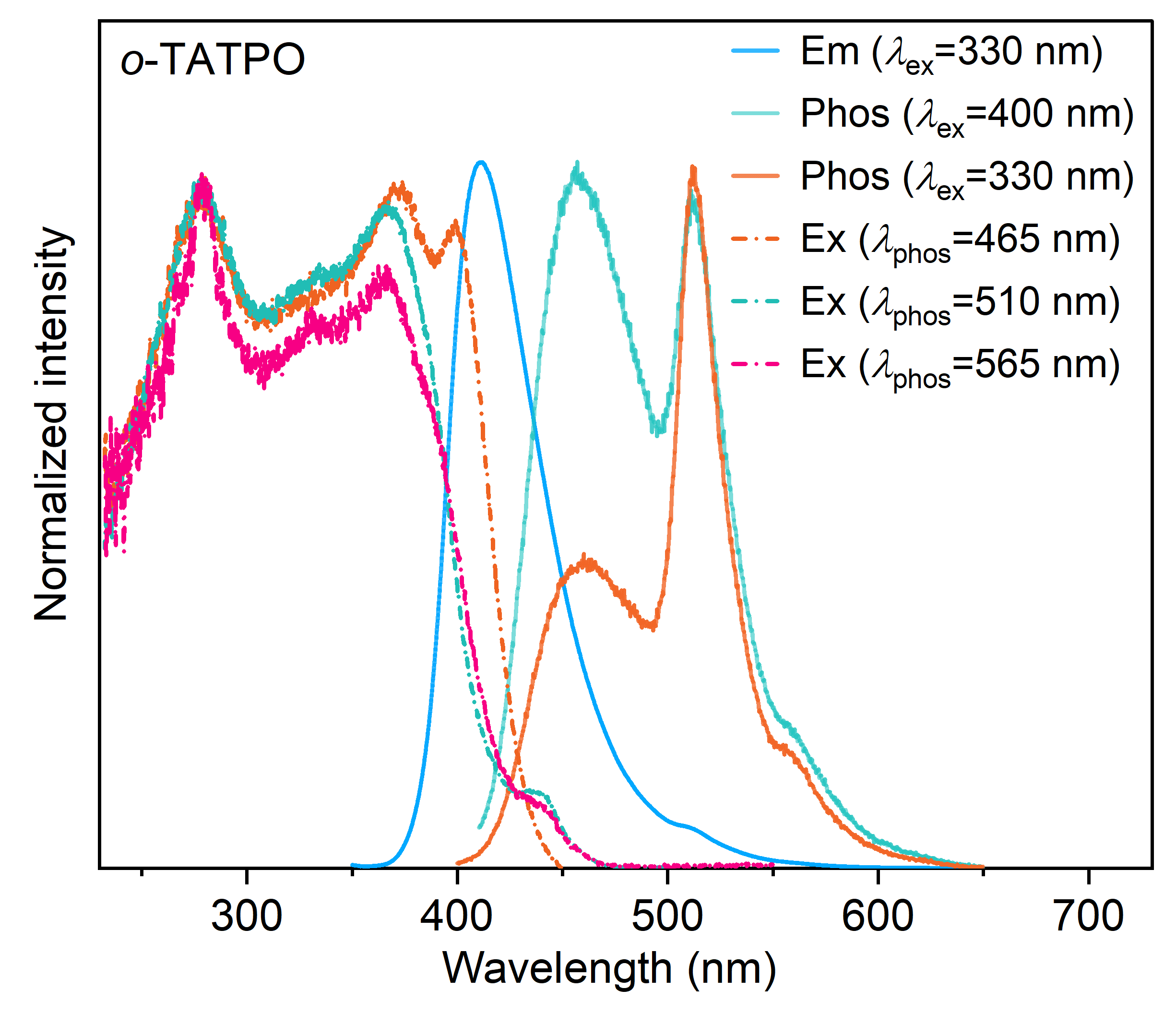


**Figure S6.** Normalized fluorescence, phosphorescence, and excitation spectra of *o*-TATPO.


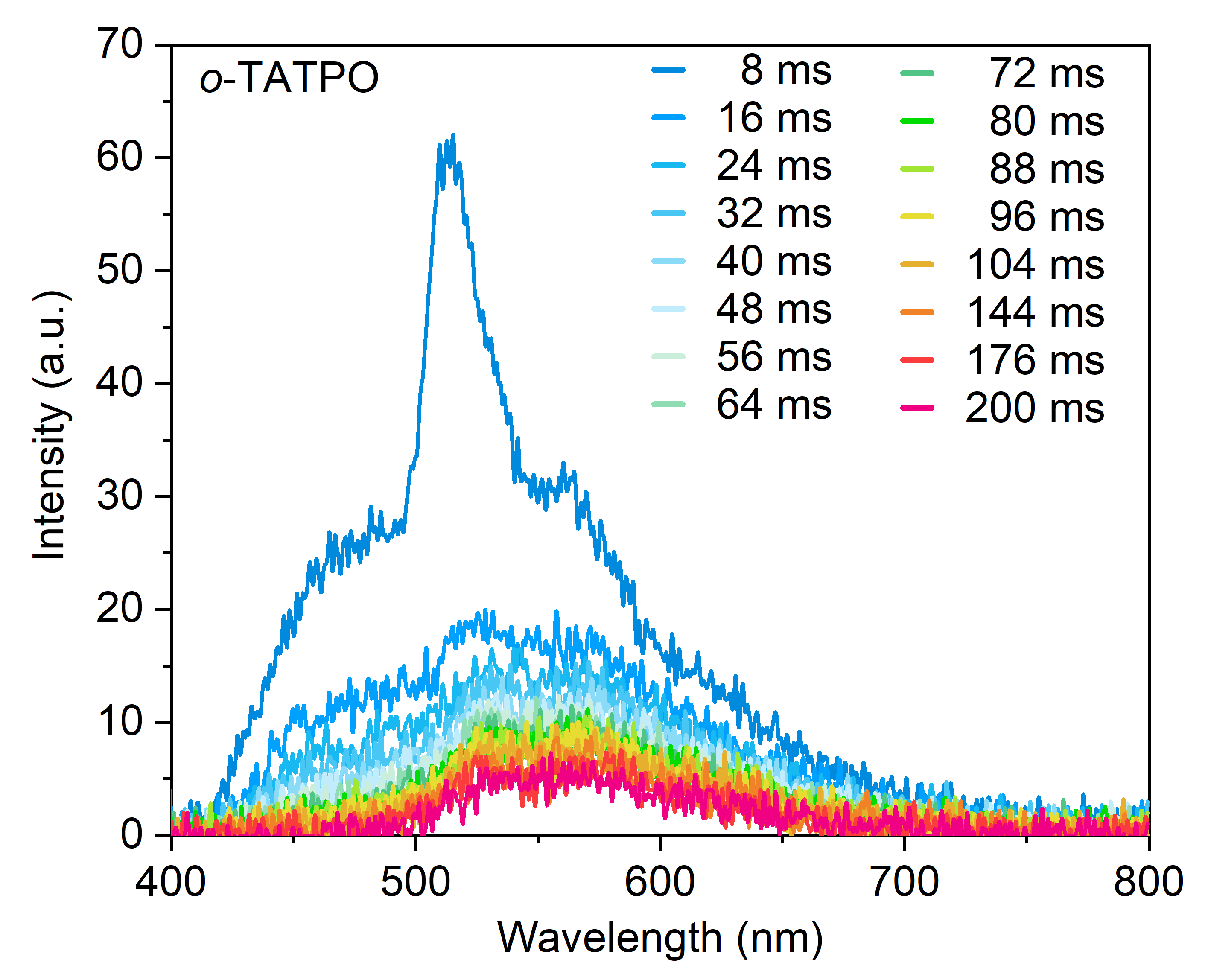


**Figure S7.** Delayed spectra of *o*-TATPO


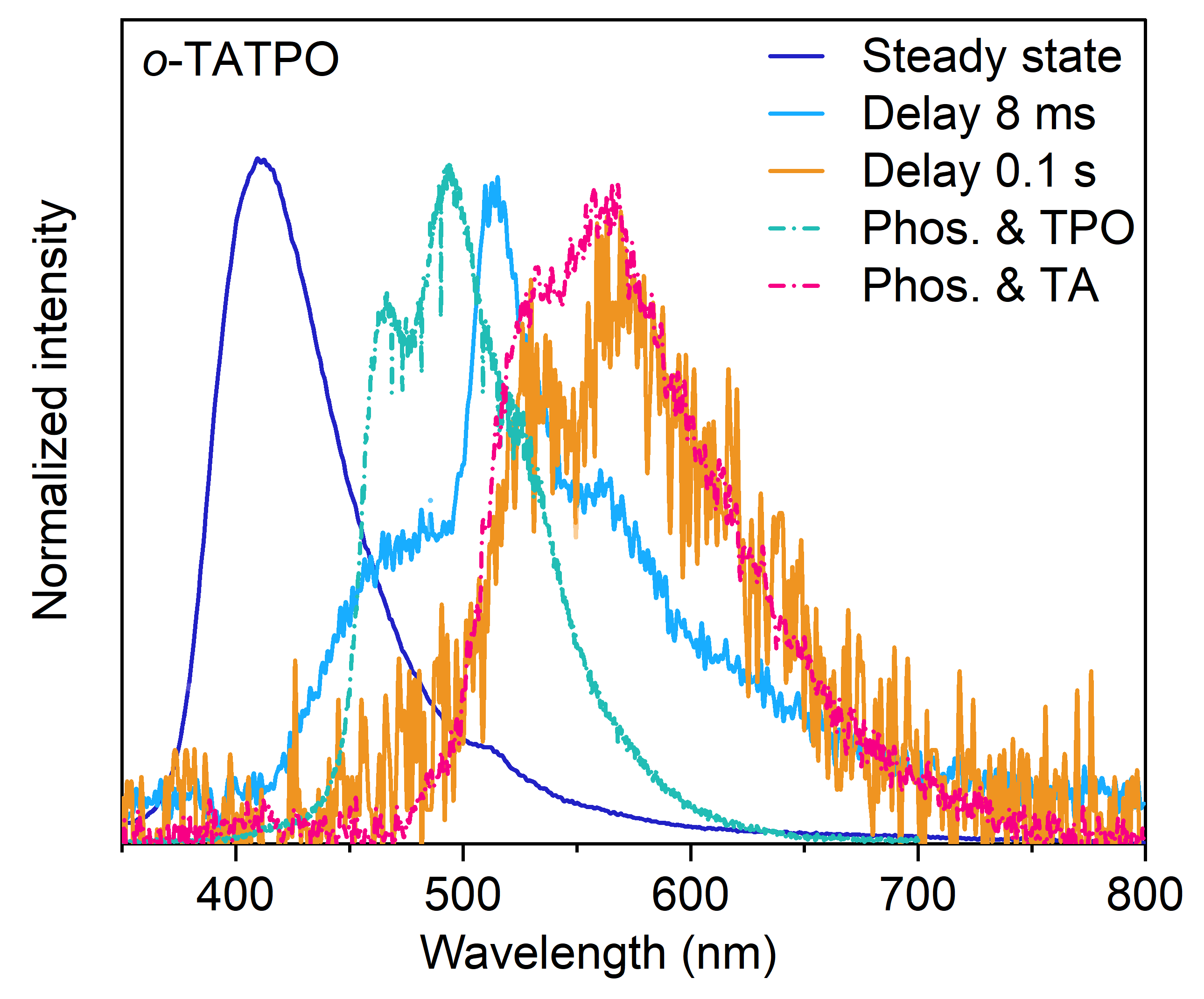


**Figure S8.** Delayed spectra of the crystalline powders of *o*-TATPO, TA, and TPO.


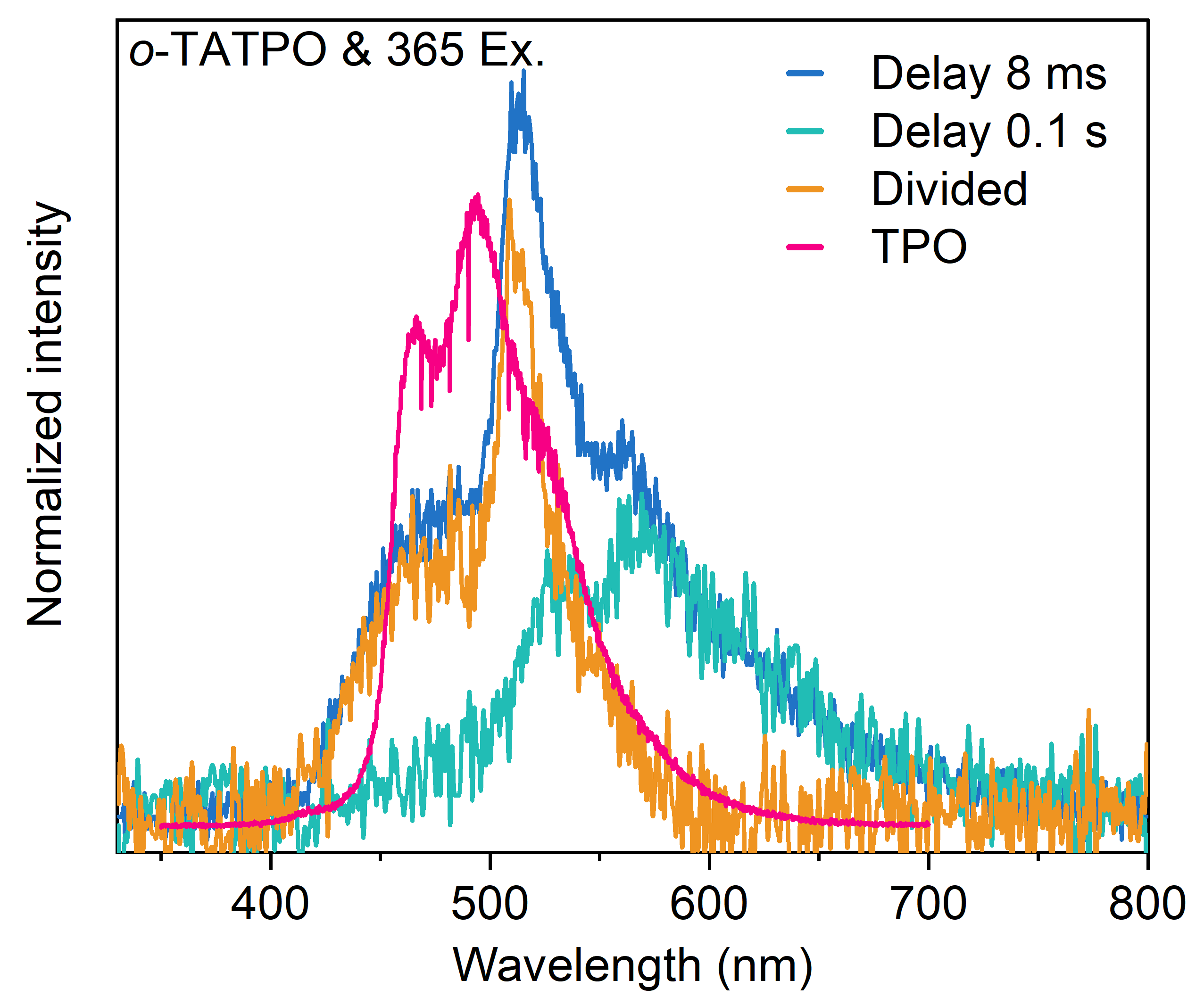


**Figure S9.** Delayed spectra of *o*-TATPO excited at 365 nm and the corresponding divided spectrum.


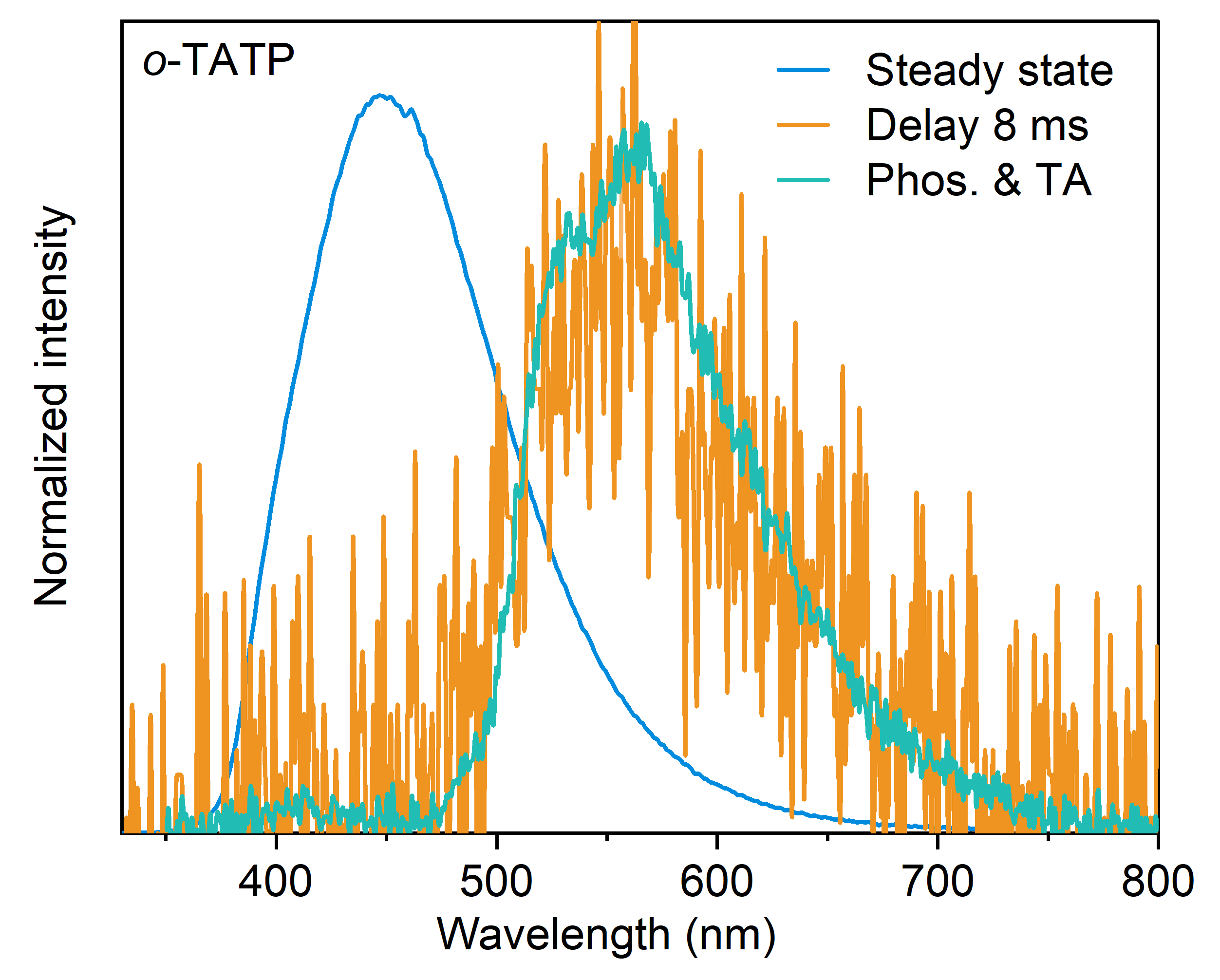


**Figure S10.** Delayed spectra of the crystalline powders of *o*-TATP and TA.


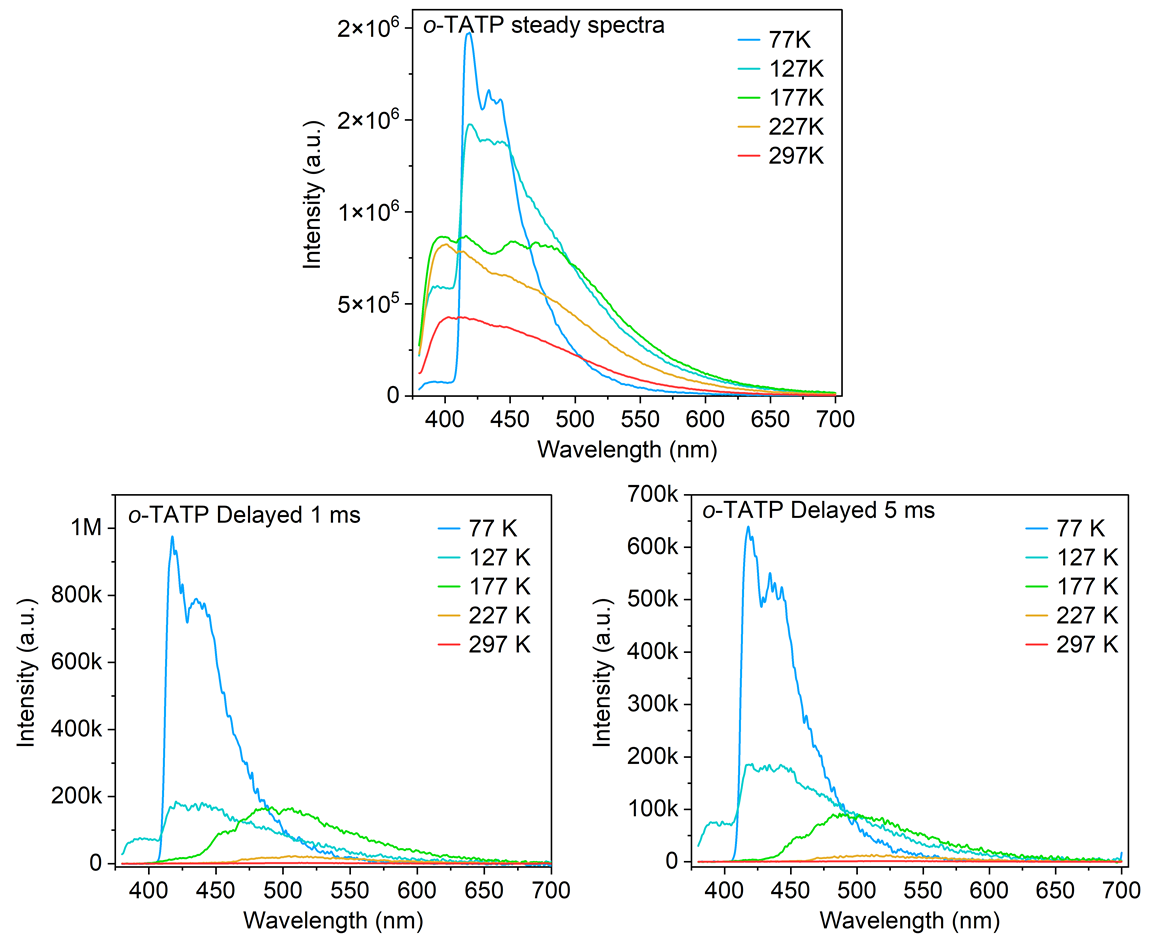


**Figure S11.** Variable-temperature steady-state and delayed spectra for crystalline *o*-TATP.


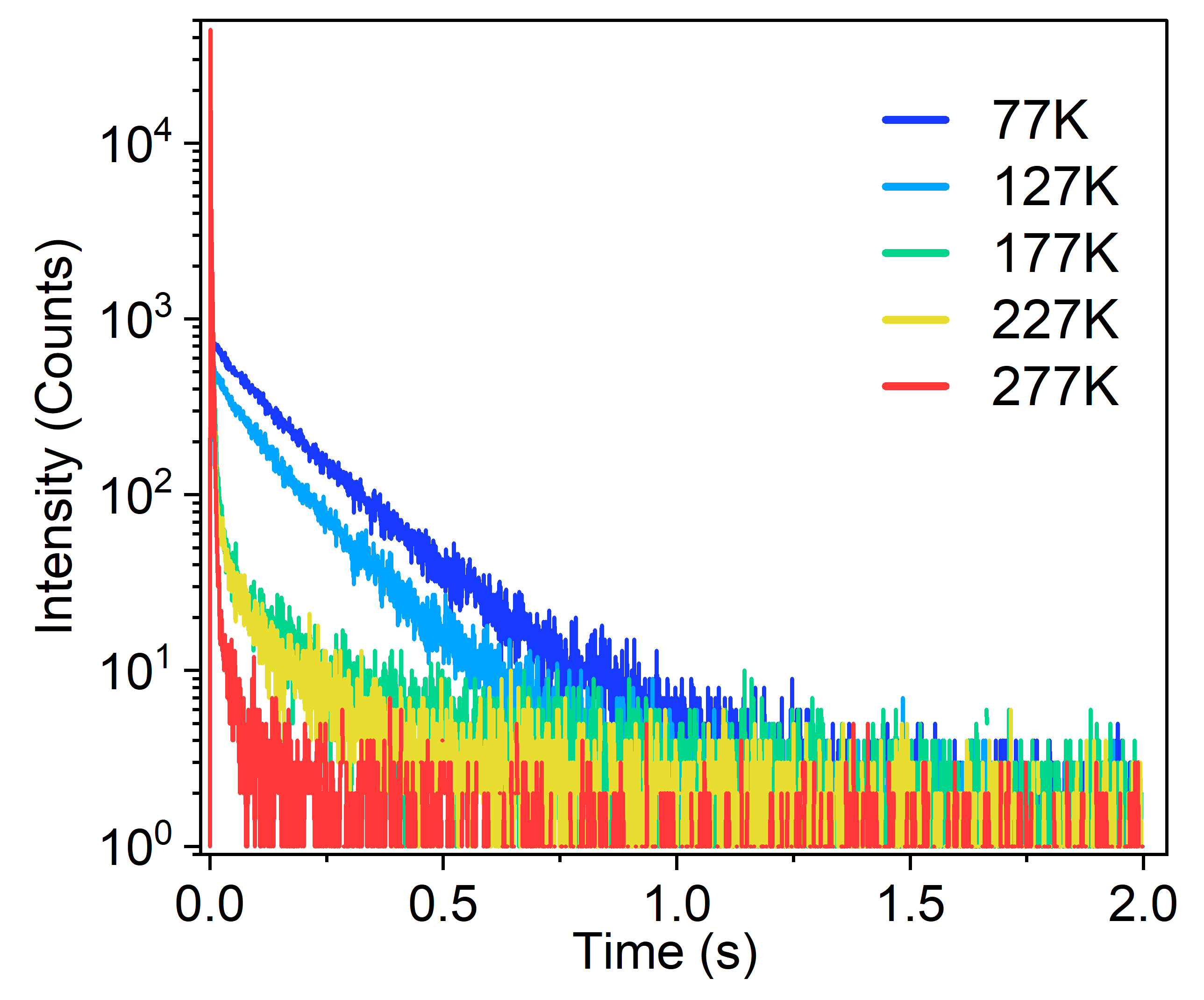


**Figure S12.** Variable-temperature time-resolved decay curves of *o*-TATPO at 465 nm.


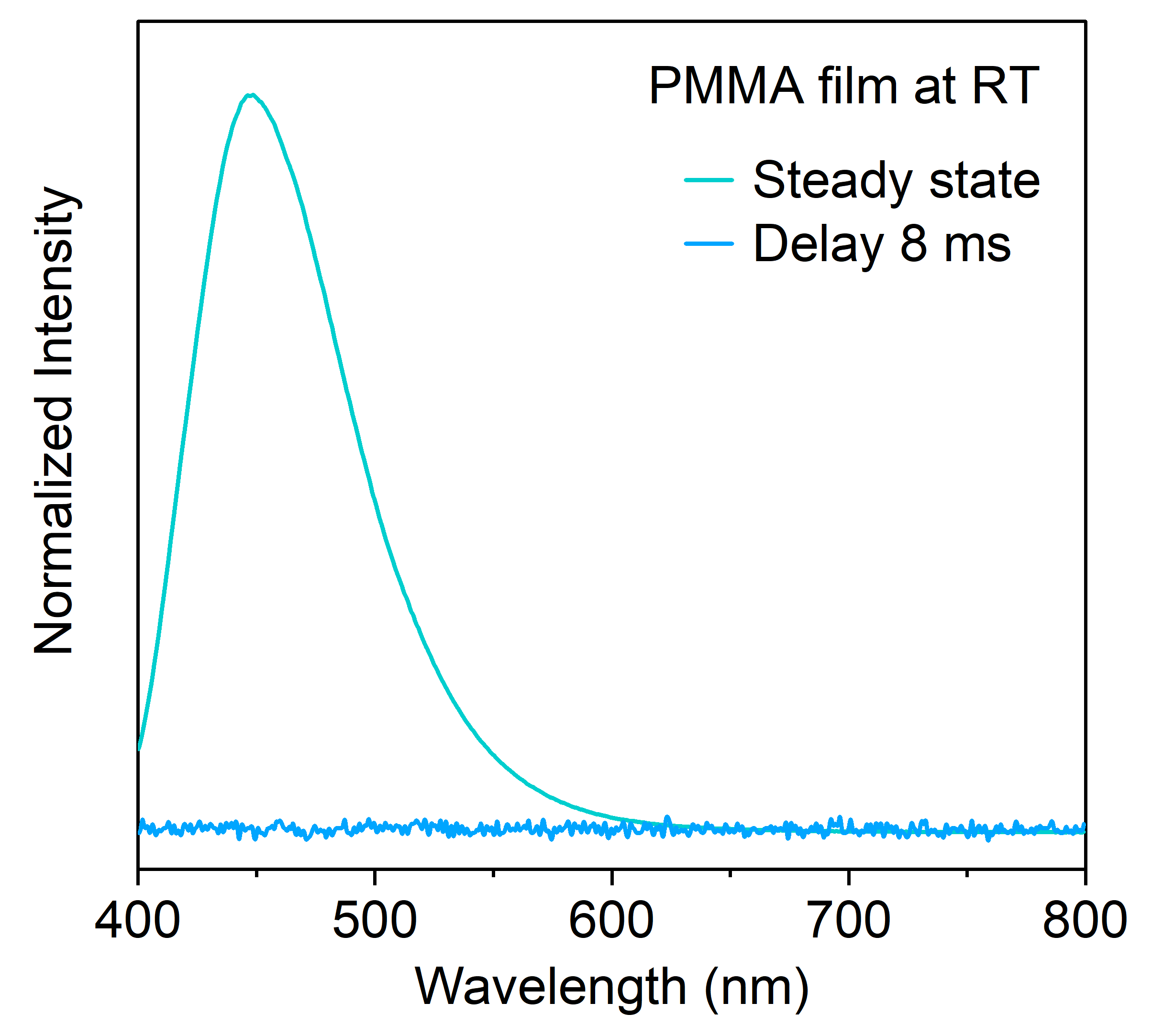


**Figure S13.** Steady-state and delayed spectra of *o*-TATPO in PMMA film at room temperature.


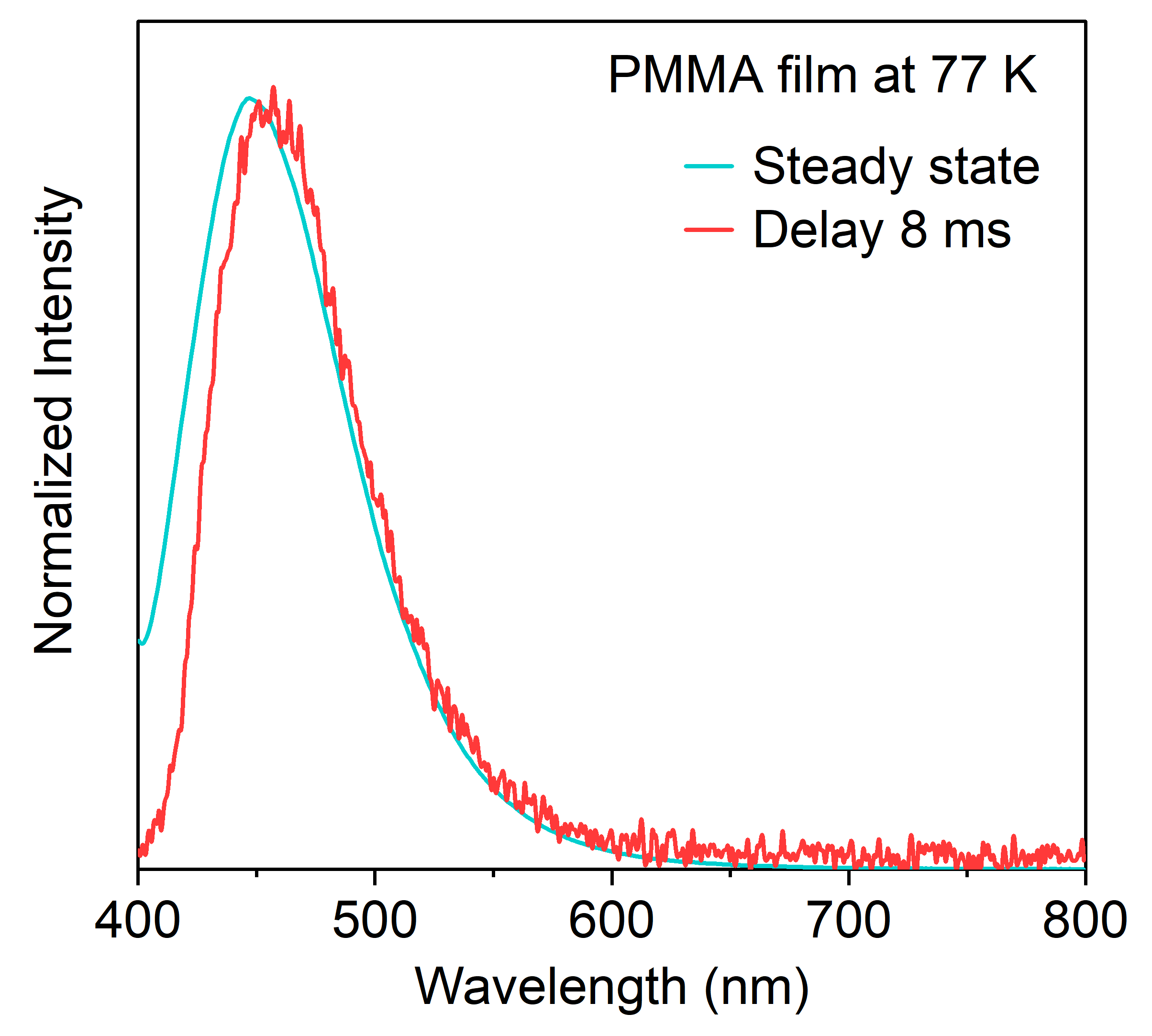


**Figure S14.** Steady-state and delayed spectra of *o*-TATPO in PMMA film at 77 K.


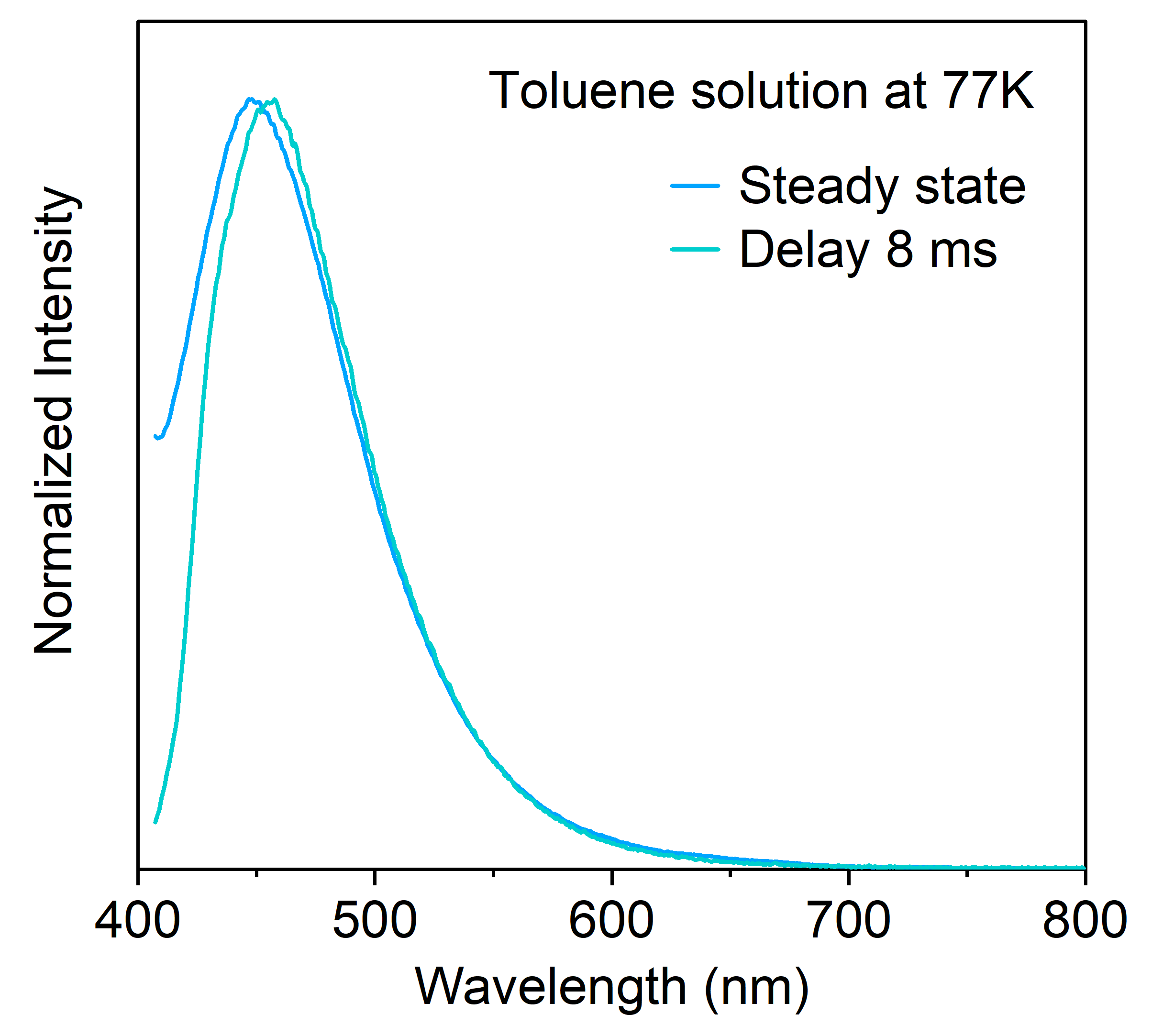


**Figure S15.** Steady-state and delayed spectra of *o*-TATPO in toluene solution (1×10^^-5^ mol·L^-1^) at 77 K.


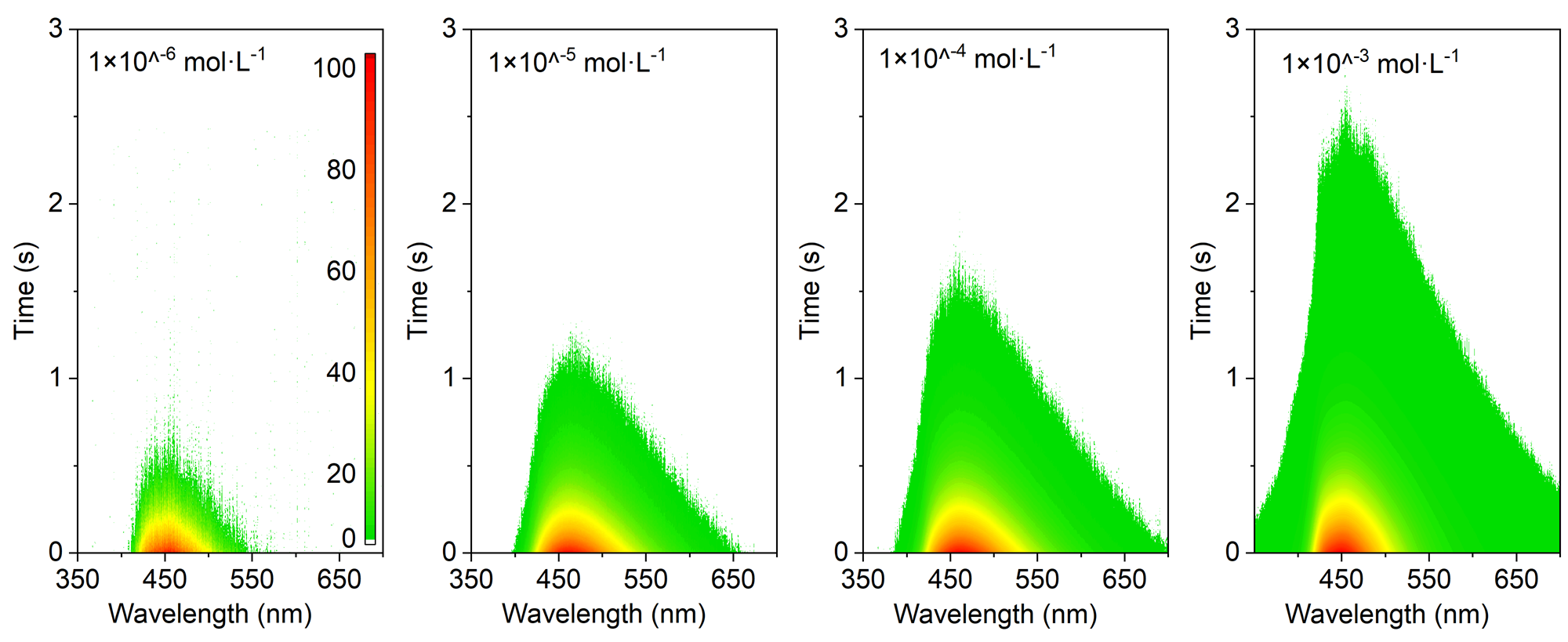


**Figure S16.** Time-resolved phosphorescence spectra for *o*-TATPO in toluene solutions with varying concentrations at 77 K.


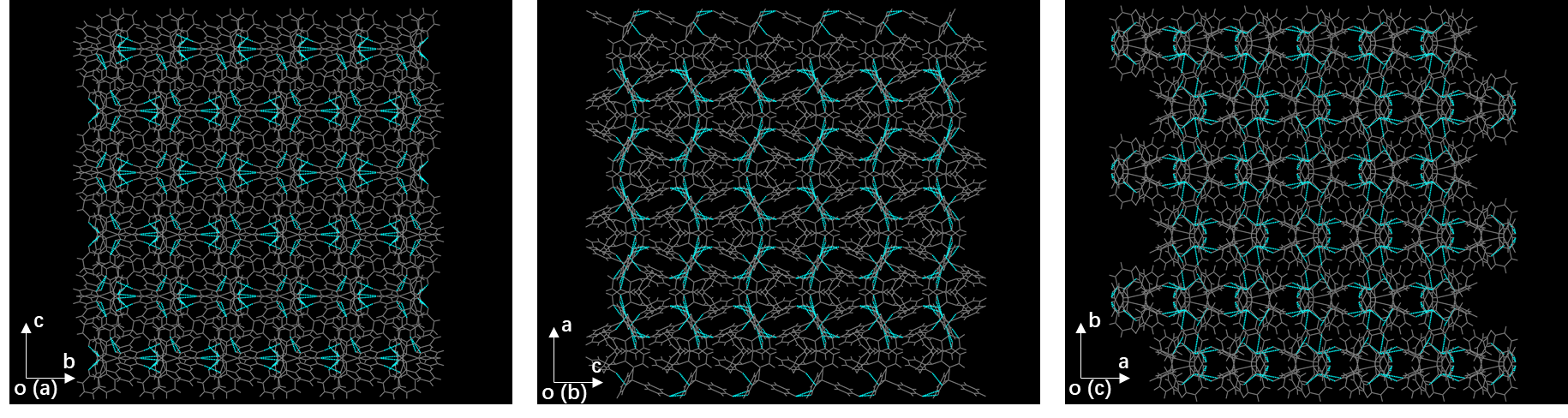


**Figure S17.** Packing patterns in the single crystals of *o*-TATPO.


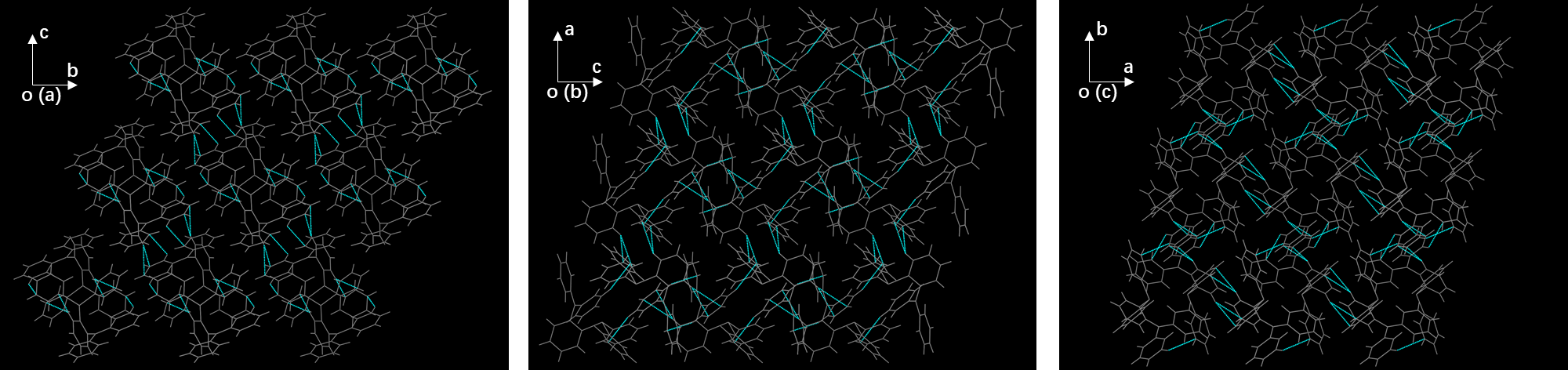


**Figure S18.** Packing patterns in the single crystals of *o*-TATP.


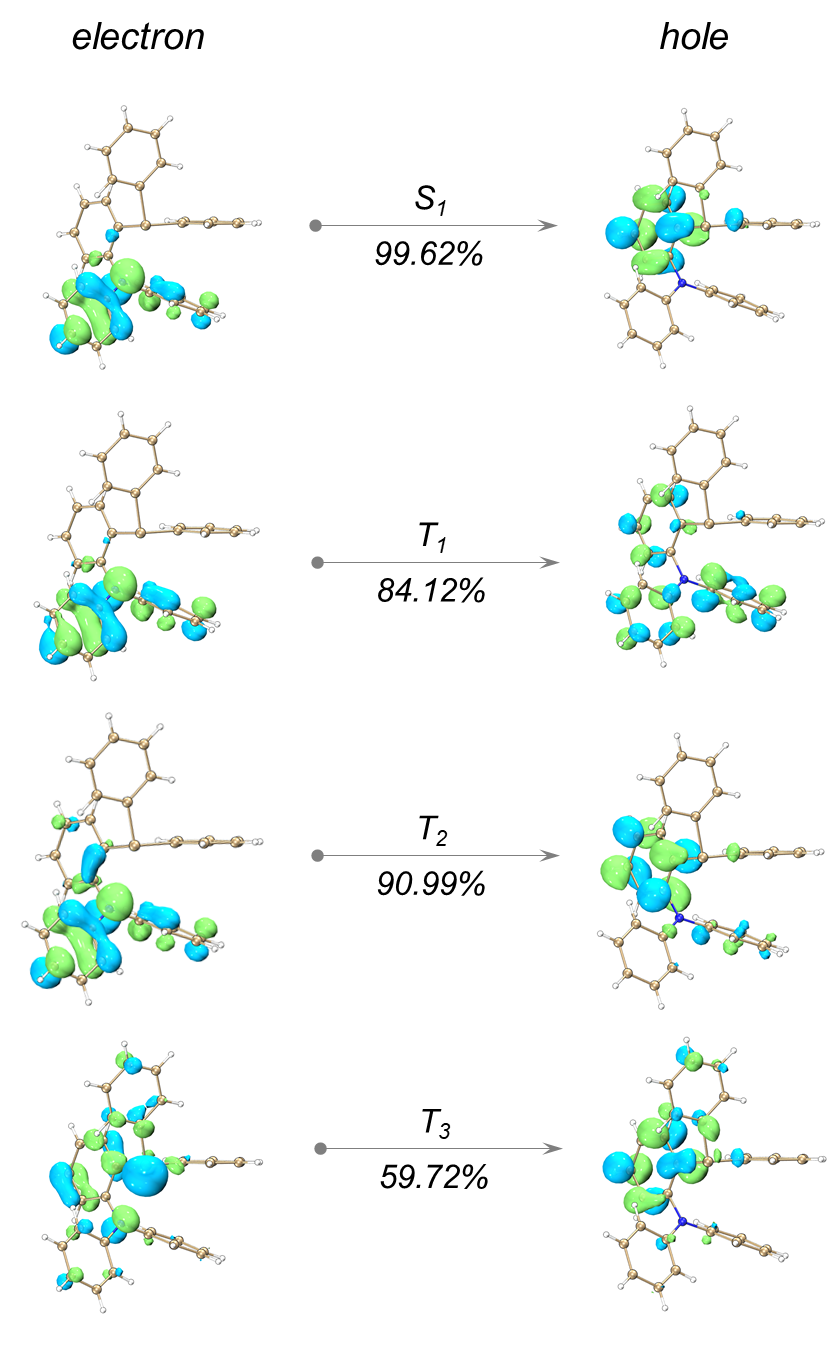


**Figure S19.** Natural transition orbitals of *o*-TATP.


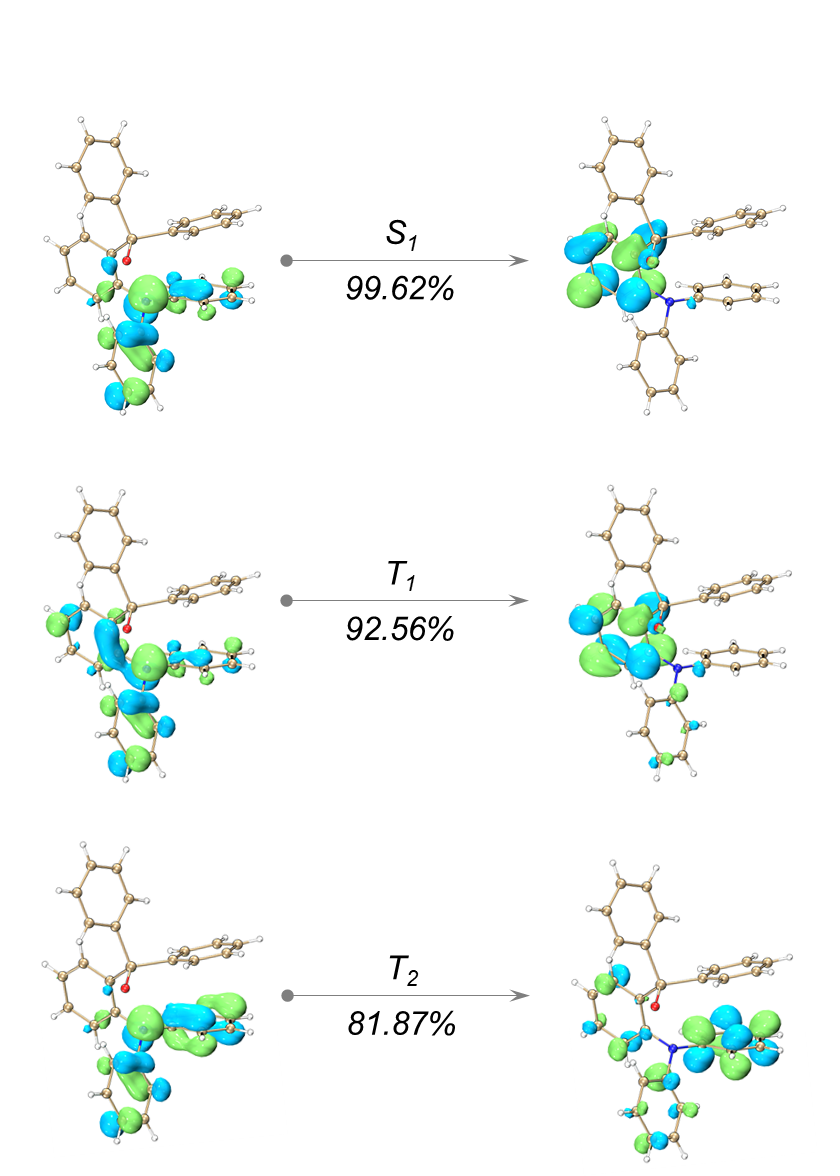


**Figure S20.** Natural transition orbitals of *o*-TATPO.

**
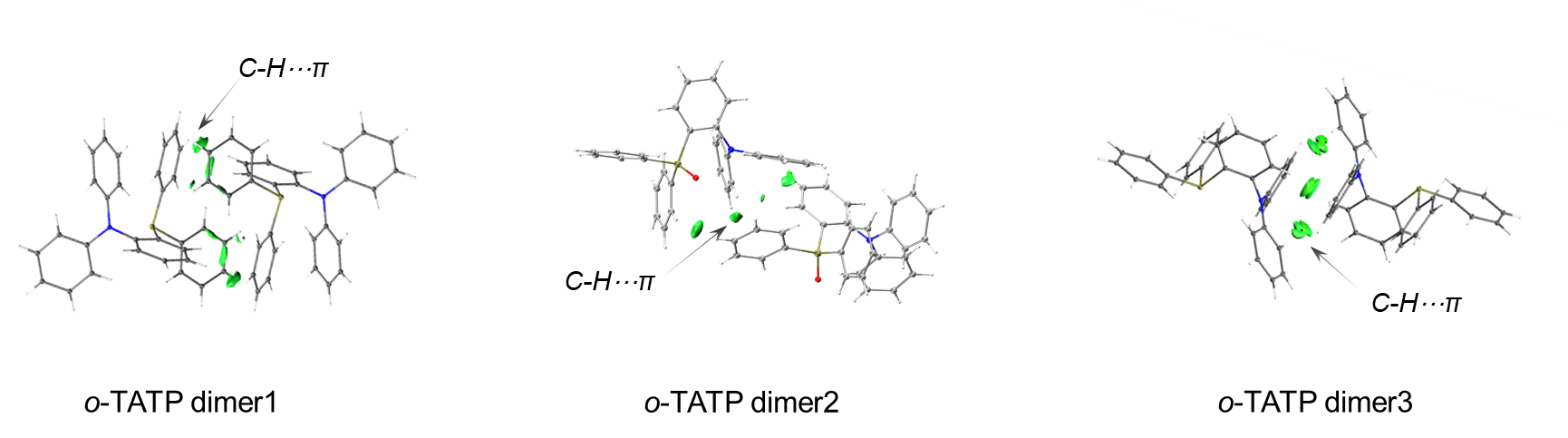
**

**Figure S21.** IGM isosurface maps in dimers of *o*-TATP with an isovalue of 0.1.

**
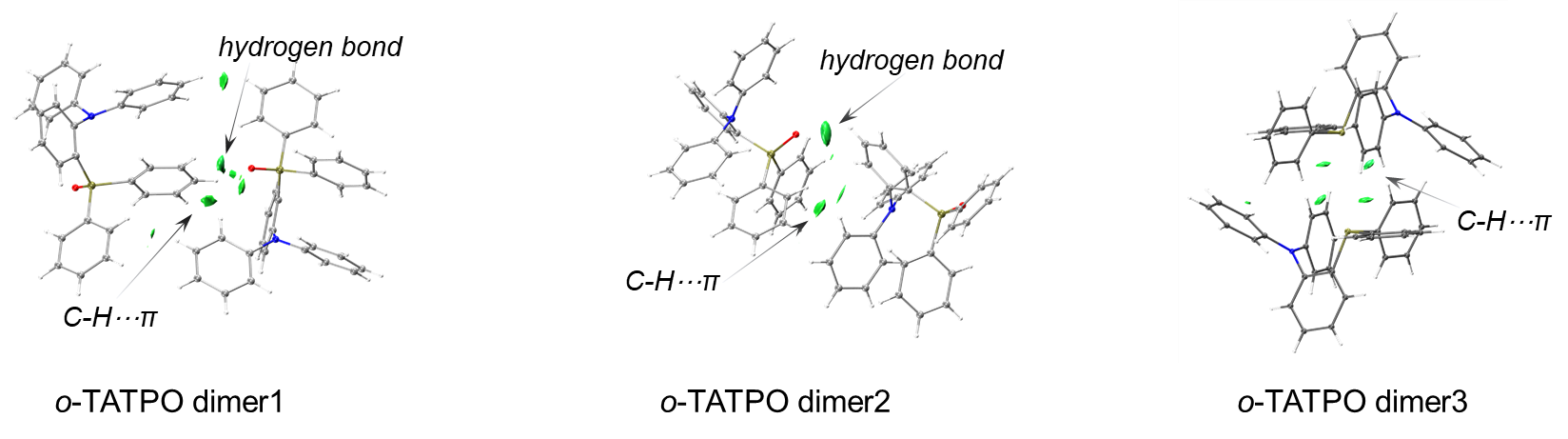
**

**Figure S22.** IGM isosurface maps in dimers of *o*-TATPO with an isovalue of 0.1.


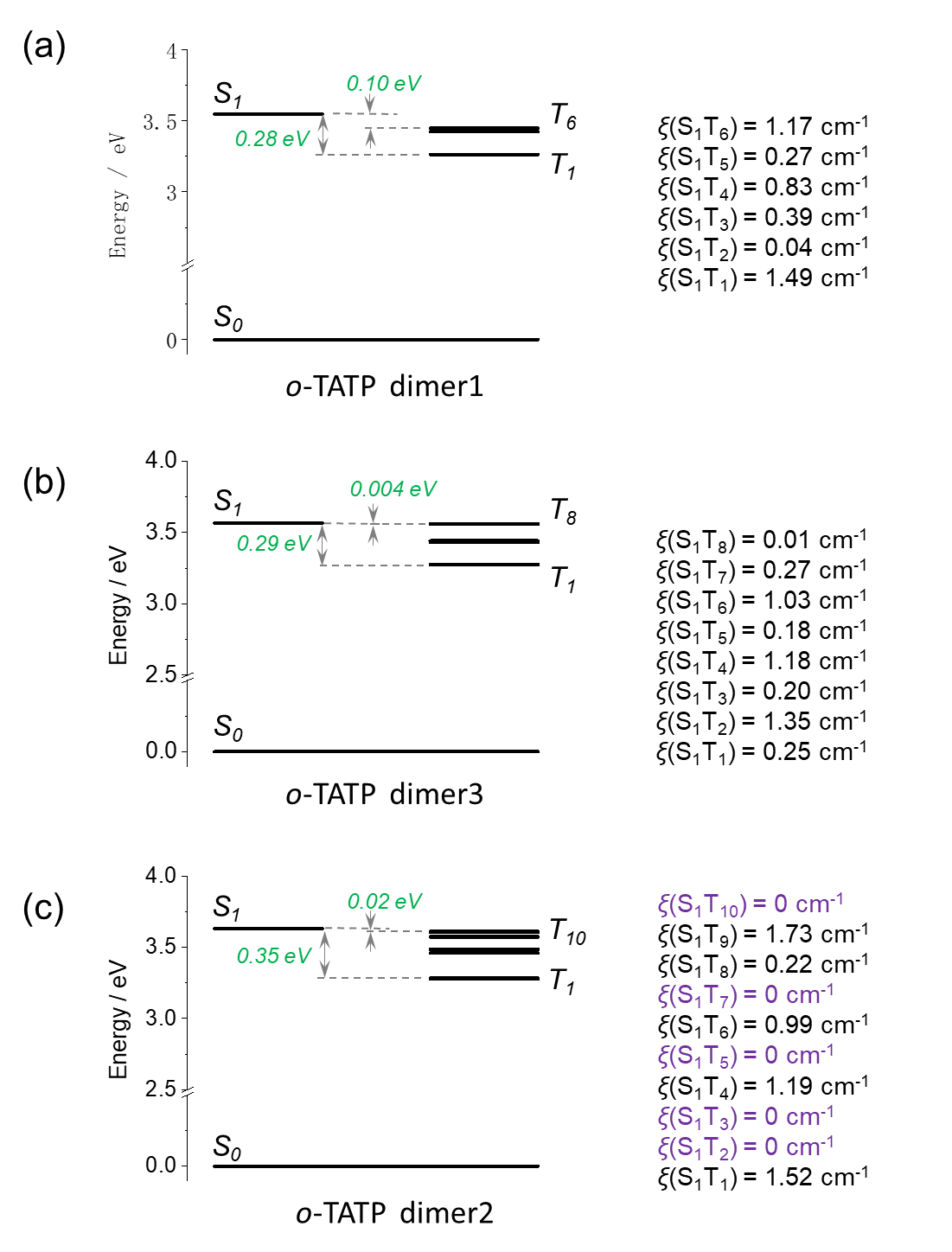


**Figure S23.** SOC matrix of the *o*-TATP dimers.


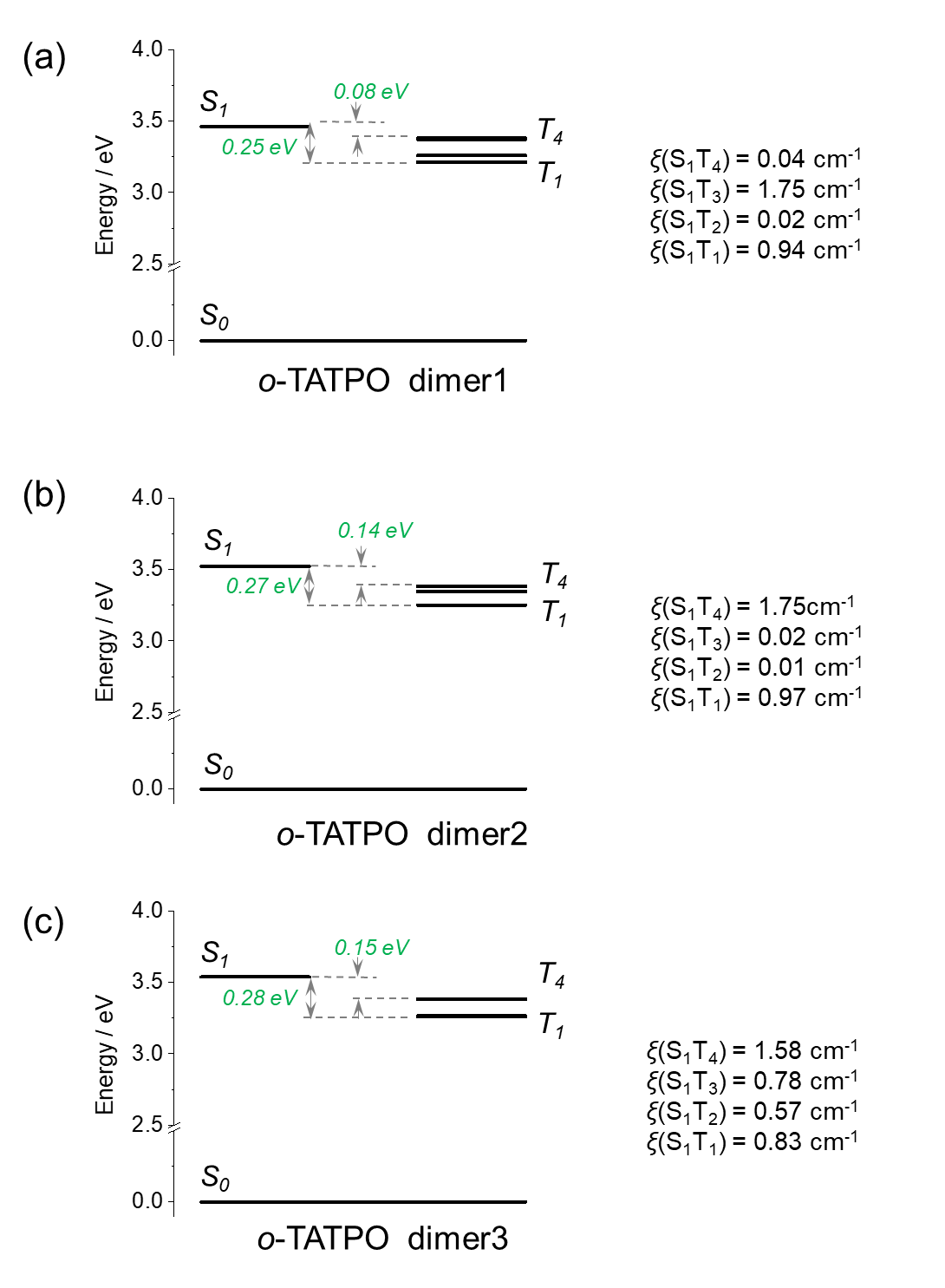


**Figure S24.** SOC matrix of the *o*-TATPO dimers.


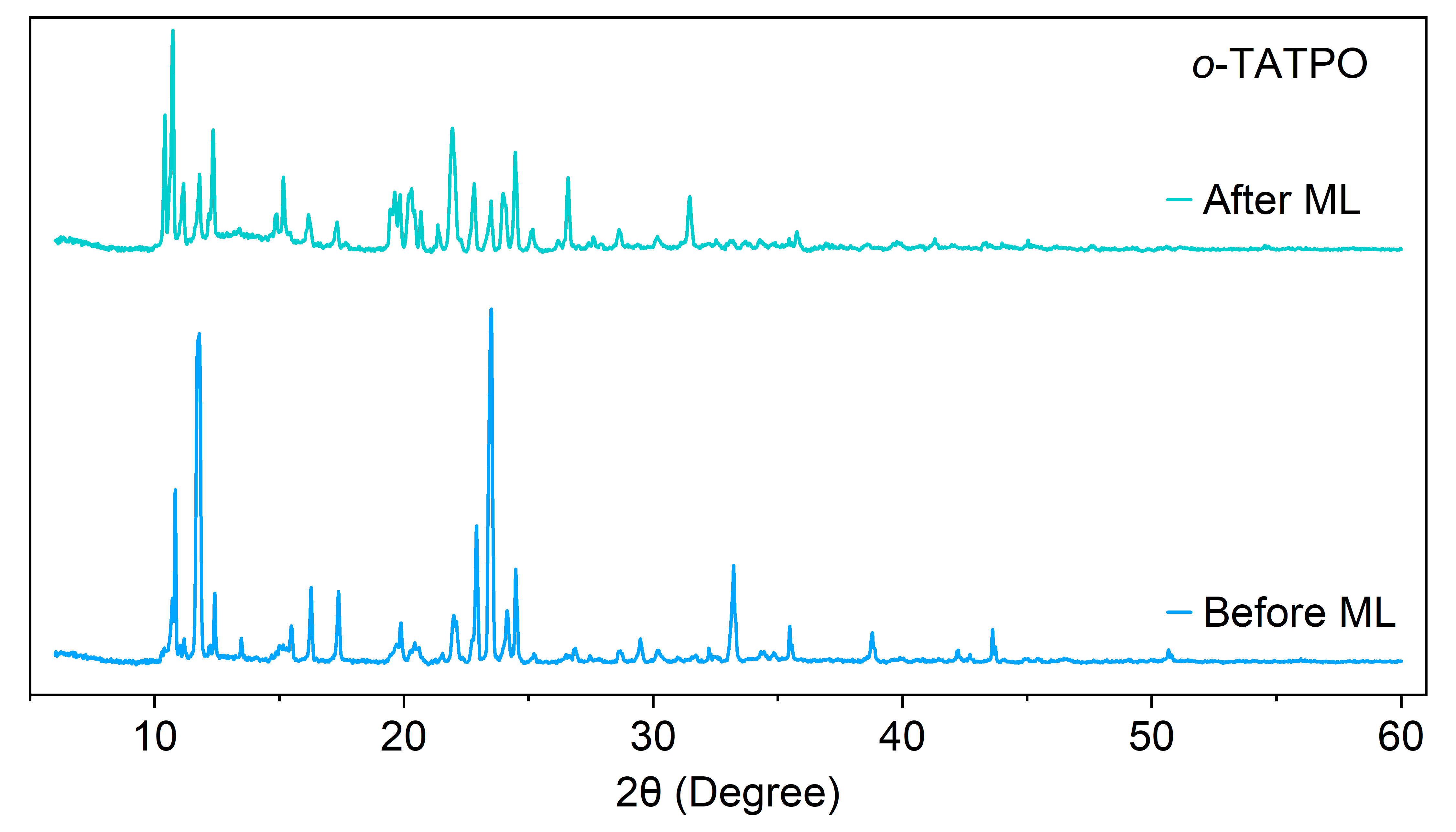


**Figure S25.** Powder X-ray diffraction (PXRD) patterns of *o*-TATPO crystalline powders before and after ML processes.

*PXRD studies for the *o*-TATPO crystalline powders before and after ML processes were carried out, as shown in Fig. S24. The PXRD data for *o*-TATPO after the ML process were similar to that before the ML process. It has been established that the crystalline structures of *o*-TATPO remained unchanged during the ML process.

**Table S1.** Photophysical properties of crystalline powders for *o*-TATP and *o*-TATPO.

| **Sample** | **Space group** | ***Φ*_PL_ (%)** | **Fluo.** | | **Phos.** | | **ML** | |
| --- | --- | --- | --- | --- | --- | --- | --- | --- |
|  |  |  | ***λ*_F_ (nm)** | ***τ*_F_ (ns)** | ***λ*_P_ (nm)** | ***τ*_P_ (ms)** |  | ***λ*_ML_ (nm)** |
| *o*-TATP | *P*-1 | 2.0 | 448 | 4.7 | 558 | 76.80 | Inactive | - |
| *o*-TATPO | *P*bca | 5.4 | 410 | 8.8 | 465; 565 | 23.24 (465 nm)  471.75 (565 nm) | Active | 450; 565 |

**Table S2.** Calculated dipole moment of *o*-TATP and *o*-TATPO based on single crystal structures.

|  | ***o*-TATP** | | | | ***o*-TATPO** | | | |
| --- | --- | --- | --- | --- | --- | --- | --- | --- |
|  | **Monomer** | **Dimer 1** | **Dimer 2** | **Dimer 3** | **Monomer** | **Dimer 1** | **Dimer 2** | **Dimer 3** |
| Dipole moment  (Debye) | 2.4603 | 0.0005 | 0.0005 | 0.0000 | 4.5158 | 6.6645 | 7.6995 | 7.9552 |

Single crystal data

**Table S3.** Crystal data and structure refinement for single crystal of *o*-TATPO.

| **Formula** | C_30_H_24_NOP | ***μ*/mm^‑1^** | 1.230 |
| --- | --- | --- | --- |
| **Formula weight** | 445.47 | **F(000)** | 1872.0 |
| **Temperature/K** | 153.0 | **Crystal size/mm^3^** | 0.5 × 0.2 × 0.1 |
| **Crystal system** | orthorhombic | **Radiation** | CuKα (l = 1.54184) |
| **Space group** | *P*bca | **Reflections collected** | 60551 |
| **a/Å** | 16.0526(6) | **Data/restraints/parameters** | 4171/0/298 |
| **b/Å** | 16.6684(6) | ***D*_x_/g cm^-3^** | 1.287 |
| **c/Å** | 17.1822(7) | **Unique (*R*_int_)** | 0.1065 |
| ***α*/°** | 90 | **Goodness-of-fit on *F*^2^** | 1.092 |
| ***β*/°** | 90 | ***R*_1_,^[a]^ *wR*_2_^[b]^ [*I≥2σ (I)*]** | *R_1_* = 0.0472, *wR_2_* = 0.1370 |
| ***γ*/°** | 90 | ***R*_1_, *wR*_2_ [all data]** | *R_1_* = 0.0776, *wR_2_* = 0.1783 |
| **Volume/Å^3^** | 4597.5(3) |  |  |
| ***ρ*_calc_/g cm^-3^** | 1.287 |  |  |

*^a^ R_1_* = Σ⎥⎥*F_o_*⎥ -⎥*Fc*⎥⎥/Σ⎥*F_o_*⎥. *^b^ wR*_2_ = [Σ[*w*(*F_o_^2^* - *F_c_^2^*)^2^]/Σ*w*(*F_o_^2^*)^2^]^1/2^, where *w* = 1/[^2^(*F_o_*)^2^ + (a*P*)^2^ + b*P*] and *P* = (*F_o_*^2^ + 2*F*_c_^2^)/3.

**Table S4.** Bond lengths for single crystal of *o*-TATPO.

| **Atom** | **Atom** | **Length/Å** | **Atom** | **Atom** | **Length/Å** |
| --- | --- | --- | --- | --- | --- |
| P1 | O1 | 1.4885(17) | C26 | C27 | 1.387(4) |
| P1 | C25 | 1.793(2) | C30 | C29 | 1.391(4) |
| P1 | C19 | 1.813(2) | C8 | C9 | 1.389(4) |
| P1 | C18 | 1.816(2) | C6 | C5 | 1.389(3) |
| N1 | C13 | 1.438(3) | C20 | C21 | 1.383(4) |
| N1 | C7 | 1.426(3) | C16 | C15 | 1.386(3) |
| N1 | C1 | 1.432(3) | C14 | C15 | 1.389(3) |
| C13 | C18 | 1.404(3) | C12 | C11 | 1.383(4) |
| C13 | C14 | 1.393(3) | C4 | C5 | 1.381(4) |
| C17 | C18 | 1.404(3) | C4 | C3 | 1.383(4) |
| C17 | C16 | 1.393(3) | C24 | C23 | 1.384(3) |
| C25 | C26 | 1.395(3) | C21 | C22 | 1.385(4) |
| C25 | C30 | 1.393(3) | C9 | C10 | 1.388(4) |
| C7 | C8 | 1.393(3) | C27 | C28 | 1.378(4) |
| C7 | C12 | 1.401(3) | C28 | C29 | 1.372(4) |
| C19 | C20 | 1.398(3) | C10 | C11 | 1.374(4) |
| C19 | C24 | 1.387(3) | C22 | C23 | 1.376(4) |
| C1 | C6 | 1.397(3) | C2 | C3 | 1.390(4) |
| C1 | C2 | 1.388(3) |  |  |  |

**Table S5.** Bond angles for single crystal of *o*-TATPO.

| **Atom** | **Atom** | **Atom** | **Angle/˚** | **Atom** | **Atom** | **Atom** | **Angle/˚** |
| --- | --- | --- | --- | --- | --- | --- | --- |
| O1 | P1 | C25 | 113.90(10) | C17 | C18 | P1 | 119.36(17) |
| O1 | P1 | C19 | 111.06(10) | C17 | C18 | C13 | 118.5(2) |
| O1 | P1 | C18 | 111.86(10) | C27 | C26 | C25 | 120.5(2) |
| C25 | P1 | C19 | 103.46(11) | C29 | C30 | C25 | 120.3(2) |
| C25 | P1 | C18 | 110.40(10) | C9 | C8 | C7 | 120.2(2) |
| C19 | P1 | C18 | 105.54(10) | C5 | C6 | C1 | 119.6(2) |
| C7 | N1 | C13 | 116.07(18) | C21 | C20 | C19 | 120.3(2) |
| C7 | N1 | C1 | 118.18(19) | C15 | C16 | C17 | 119.6(2) |
| C1 | N1 | C13 | 116.79(18) | C15 | C14 | C13 | 120.9(2) |
| C18 | C13 | N1 | 120.2(2) | C11 | C12 | C7 | 119.8(2) |
| C14 | C13 | N1 | 119.9(2) | C5 | C4 | C3 | 119.1(2) |
| C14 | C13 | C18 | 119.9(2) | C23 | C24 | C19 | 120.6(2) |
| C16 | C17 | C18 | 121.2(2) | C20 | C21 | C22 | 119.9(2) |
| C26 | C25 | P1 | 124.08(19) | C10 | C9 | C8 | 120.5(2) |
| C30 | C25 | P1 | 116.69(18) | C28 | C27 | C26 | 119.6(3) |
| C30 | C25 | C26 | 118.9(2) | C16 | C15 | C14 | 119.9(2) |
| C8 | C7 | N1 | 122.2(2) | C29 | C28 | C27 | 121.0(2) |
| C8 | C7 | C12 | 119.0(2) | C11 | C10 | C9 | 119.2(2) |
| C12 | C7 | N1 | 118.8(2) | C10 | C11 | C12 | 121.3(2) |
| C20 | C19 | P1 | 118.27(18) | C28 | C29 | C30 | 119.7(2) |
| C24 | C19 | P1 | 122.88(18) | C23 | C22 | C21 | 120.2(2) |
| C24 | C19 | C20 | 118.9(2) | C1 | C2 | C3 | 120.2(2) |
| C6 | C1 | N1 | 118.4(2) | C22 | C23 | C24 | 120.0(3) |
| C2 | C1 | N1 | 122.2(2) | C4 | C5 | C6 | 121.1(2) |
| C2 | C1 | C6 | 119.3(2) | C4 | C3 | C2 | 120.7(3) |
| C13 | C18 | P1 | 120.92(17) |  |  |  |  |


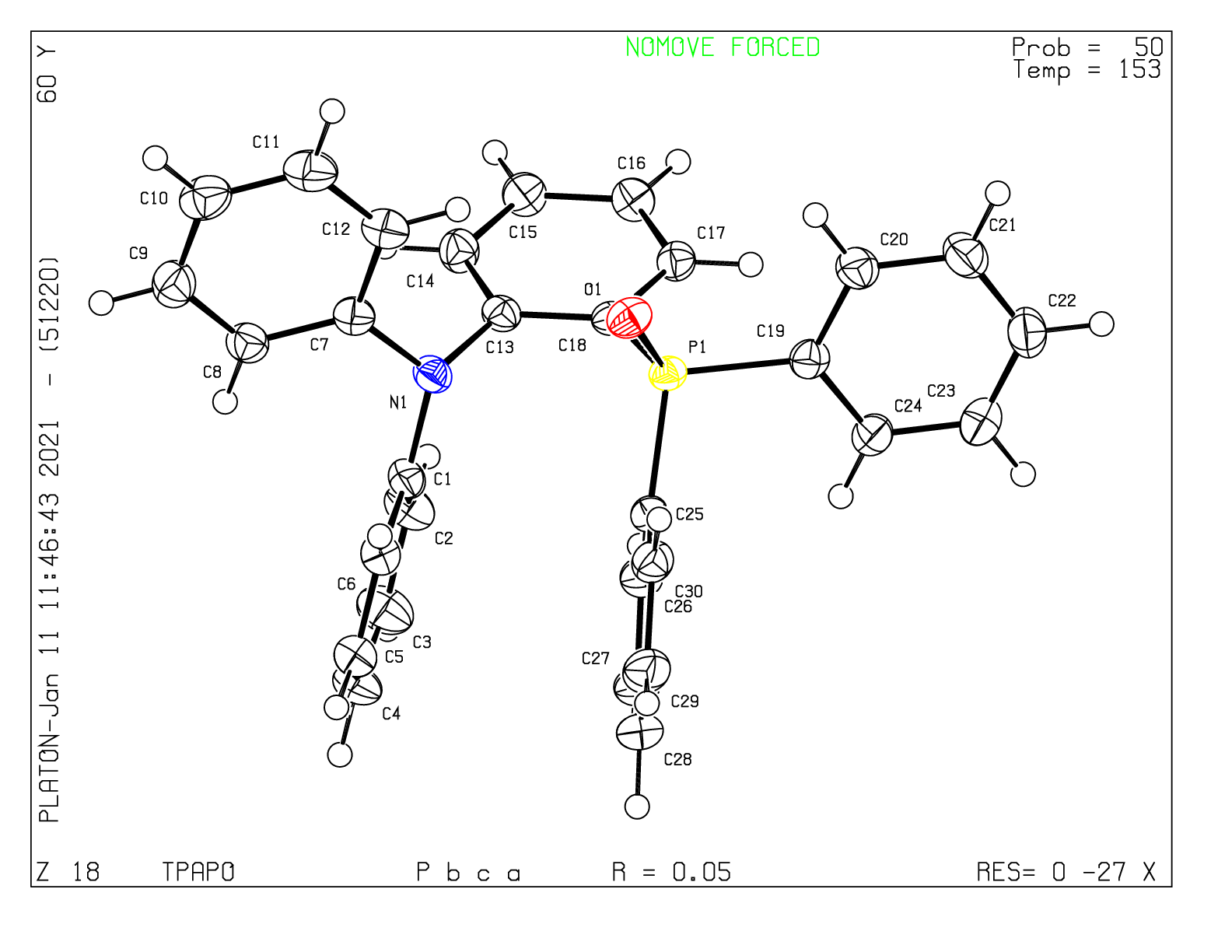


**Figure S26.** Single crystal structure of *o*-TATPO.

**Table S6.** Crystal data and structure refinement for single crystal of *o*-TATP.

| **Formula** | C_30_H_24_NOP | ***μ*/mm^‑1^** | 1.207 |
| --- | --- | --- | --- |
| **Formula weight** | 429.47 | **F(000)** | 452.0 |
| **Temperature/K** | 153.0 | **Crystal size/mm^3^** | 0.5 × 0.4 × 0.2 |
| **Crystal system** | triclinic | **Radiation** | CuKα (l = 1.54184) |
| **Space group** | *P*-1 | **Reflections collected** | 18985 |
| **a/Å** | 10.3145(16) | **Data/restraints/parameters** | 3863/0/290 |
| **b/Å** | 11.0107(17) | ***D*_x_/g cm^-3^** | 1.272 |
| **c/Å** | 11.2287(18) | **Unique (*R*_int_)** | 0.0495 |
| ***α*/°** | 111.844(4) | **Goodness-of-fit on *F*^2^** | 1.133 |
| ***β*/°** | 101.995(4) | ***R*_1_,^[a]^ *wR*_2_^[b]^ [*I≥2σ (I)*]** | *R_1_* = 0.0549, *wR_2_* = 0.1764 |
| ***γ*/°** | 98.621(4) | ***R*_1_, *wR*_2_ [all data]** | *R_1_* = 0.1043, *wR_2_* = 0.2233 |
| **Volume/Å^3^** | 1121.0(3) |  |  |
| ***ρ*_calc_/g cm^-3^** | 1.272 |  |  |

*^a^ R_1_* = Σ⎥⎥*F_o_*⎥ -⎥*Fc*⎥⎥/Σ⎥*F_o_*⎥. *^b^ wR*_2_ = [Σ[*w*(*F_o_^2^* - *F_c_^2^*)^2^]/Σ*w*(*F_o_^2^*)^2^]^1/2^, where *w* = 1/[^2^(*F_o_*)^2^ + (a*P*)^2^ + b*P*] and *P* = (*F_o_*^2^ + 2*F*_c_^2^)/3.

**Table S7.** Bond lengths for single crystal of *o*-TATP.

| **Atom** | **Atom** | **Length/Å** | **Atom** | **Atom** | **Length/Å** |
| --- | --- | --- | --- | --- | --- |
| P1 | C19 | 1.845(3) | C30 | C25 | 1.392(4) |
| P1 | C25 | 1.834(3) | C30 | C29 | 1.389(4) |
| P1 | C18 | 1.839(3) | C17 | C18 | 1.397(4) |
| N1 | C13 | 1.438(3) | C24 | C23 | 1.399(5) |
| N1 | C7 | 1.409(3) | C14 | C15 | 1.396(4) |
| N1 | C1 | 1.429(3) | C1 | C2 | 1.392(4) |
| C26 | C25 | 1.383(4) | C1 | C6 | 1.386(4) |
| C26 | C27 | 1.392(4) | C27 | C28 | 1.377(4) |
| C19 | C24 | 1.392(4) | C2 | C3 | 1.394(4) |
| C19 | C20 | 1.402(4) | C28 | C29 | 1.374(5) |
| C8 | C7 | 1.405(4) | C23 | C22 | 1.382(4) |
| C8 | C9 | 1.395(4) | C20 | C21 | 1.383(5) |
| C13 | C14 | 1.381(4) | C6 | C5 | 1.391(4) |
| C13 | C18 | 1.396(4) | C4 | C3 | 1.374(4) |
| C12 | C7 | 1.400(4) | C4 | C5 | 1.391(4) |
| C12 | C11 | 1.393(4) | C21 | C22 | 1.392(4) |
| C16 | C17 | 1.385(4) | C9 | C10 | 1.381(4) |
| C16 | C15 | 1.385(4) | C11 | C10 | 1.386(5) |
| P1 | C19 | 1.845(3) | C30 | C25 | 1.392(4) |

**Table S8.** Bond angles for single crystal of o-TATP.

| **Atom** | **Atom** | **Atom** | **Angle/˚** | **Atom** | **Atom** | **Atom** | **Angle/˚** |
| --- | --- | --- | --- | --- | --- | --- | --- |
| C25 | P1 | C19 | 102.69(12) | C30 | C25 | P1 | 117.6(2) |
| C25 | P1 | C18 | 102.51(12) | C13 | C14 | C15 | 119.5(3) |
| C18 | P1 | C19 | 100.55(13) | C13 | C18 | P1 | 117.8(2) |
| C7 | N1 | C13 | 119.5(2) | C13 | C18 | C17 | 118.7(2) |
| C7 | N1 | C1 | 123.4(2) | C17 | C18 | P1 | 123.5(2) |
| C1 | N1 | C13 | 116.5(2) | C2 | C1 | N1 | 119.3(2) |
| C25 | C26 | C27 | 120.5(3) | C6 | C1 | N1 | 121.9(2) |
| C24 | C19 | P1 | 124.6(2) | C6 | C1 | C2 | 118.7(2) |
| C24 | C19 | C20 | 118.1(3) | C16 | C15 | C14 | 120.2(2) |
| C20 | C19 | P1 | 117.2(2) | C28 | C27 | C26 | 120.6(3) |
| C9 | C8 | C7 | 119.8(3) | C1 | C2 | C3 | 120.8(3) |
| C14 | C13 | N1 | 119.2(2) | C29 | C28 | C27 | 119.3(3) |
| C14 | C13 | C18 | 121.0(3) | C22 | C23 | C24 | 120.8(3) |
| C18 | C13 | N1 | 119.8(2) | C21 | C20 | C19 | 121.2(3) |
| C11 | C12 | C7 | 120.5(3) | C1 | C6 | C5 | 120.2(3) |
| C15 | C16 | C17 | 119.9(3) | C3 | C4 | C5 | 119.2(3) |
| C29 | C30 | C25 | 120.5(3) | C20 | C21 | C22 | 120.4(3) |
| C8 | C7 | N1 | 122.0(2) | C10 | C9 | C8 | 121.4(3) |
| C12 | C7 | N1 | 119.5(3) | C4 | C3 | C2 | 120.3(3) |
| C12 | C7 | C8 | 118.5(2) | C28 | C29 | C30 | 120.5(3) |
| C16 | C17 | C18 | 120.7(3) | C6 | C5 | C4 | 120.7(3) |
| C19 | C24 | C23 | 120.5(3) | C23 | C22 | C21 | 119.0(3) |
| C26 | C25 | P1 | 123.8(2) | C10 | C11 | C12 | 120.8(3) |
| C26 | C25 | C30 | 118.5(2) | C9 | C10 | C11 | 118.9(3) |
| C25 | P1 | C19 | 102.69(12) | C30 | C25 | P1 | 117.6(2) |
| C25 | P1 | C18 | 102.51(12) | C13 | C14 | C15 | 119.5(3) |


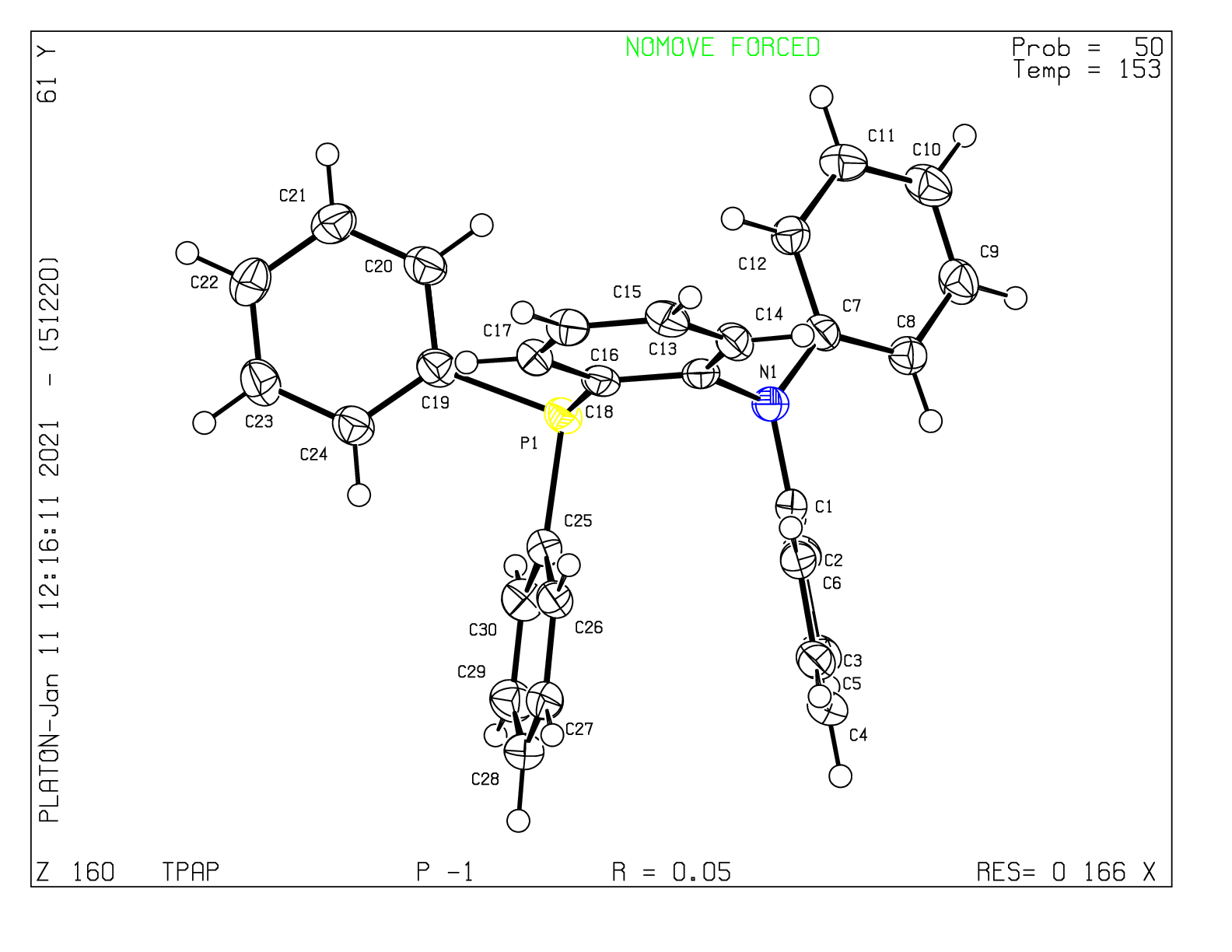


**Figure S27.** Single crystal structure of *o*-TATP.

Characterization of chemical structure

**Figure S28.** ^1^H NMR spectrum of *o*-TATP (in DMSO-*d_6_*).


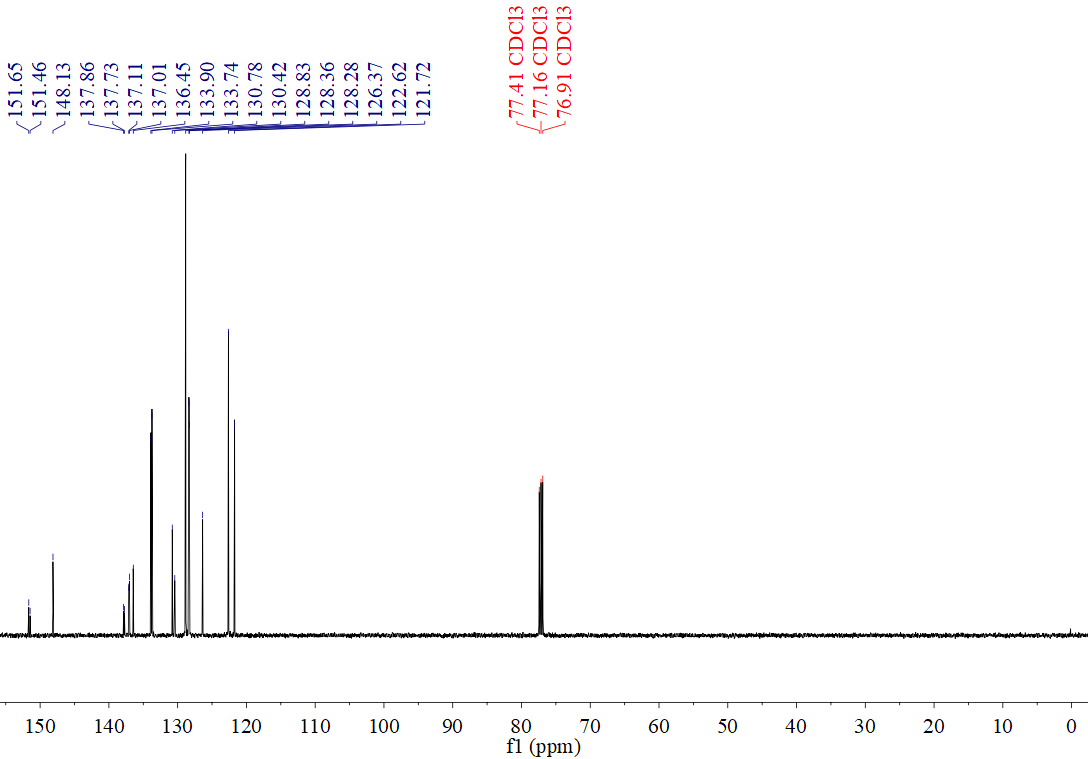


**Figure S29.** ^13^C NMR spectrum of *o*-TATP (in Chloroform-*d*).


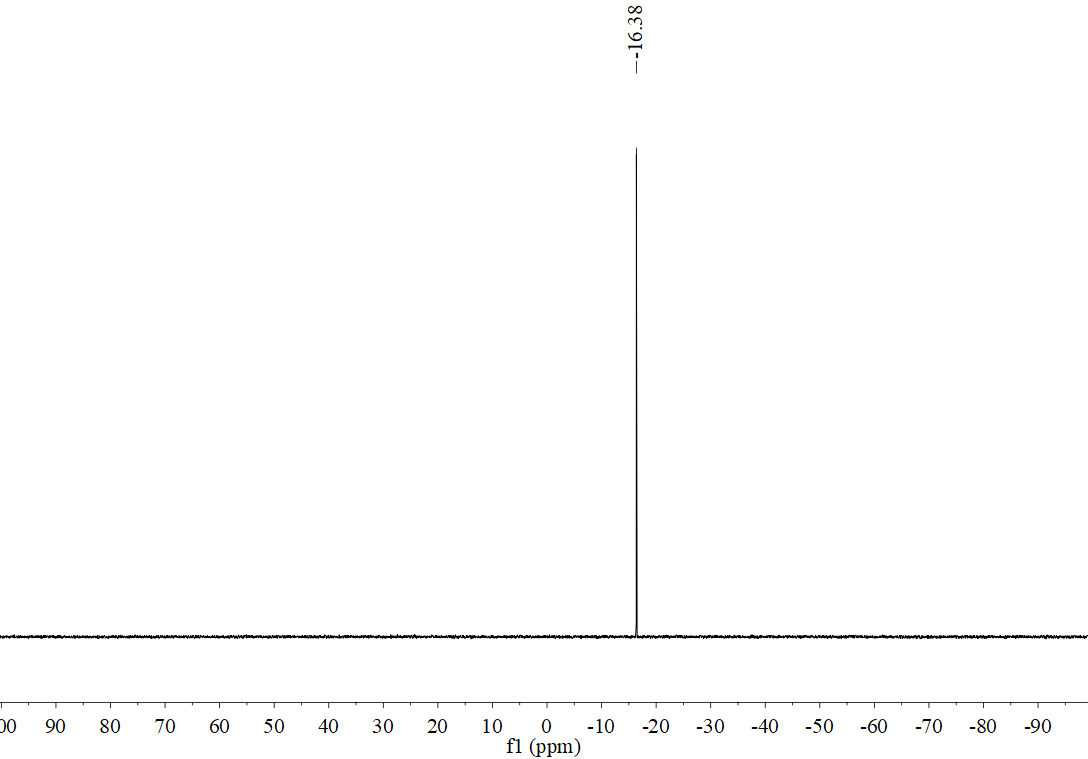


**Figure S30.** ^31^P NMR spectrum of *o*-TATP (in Chloroform-*d*).


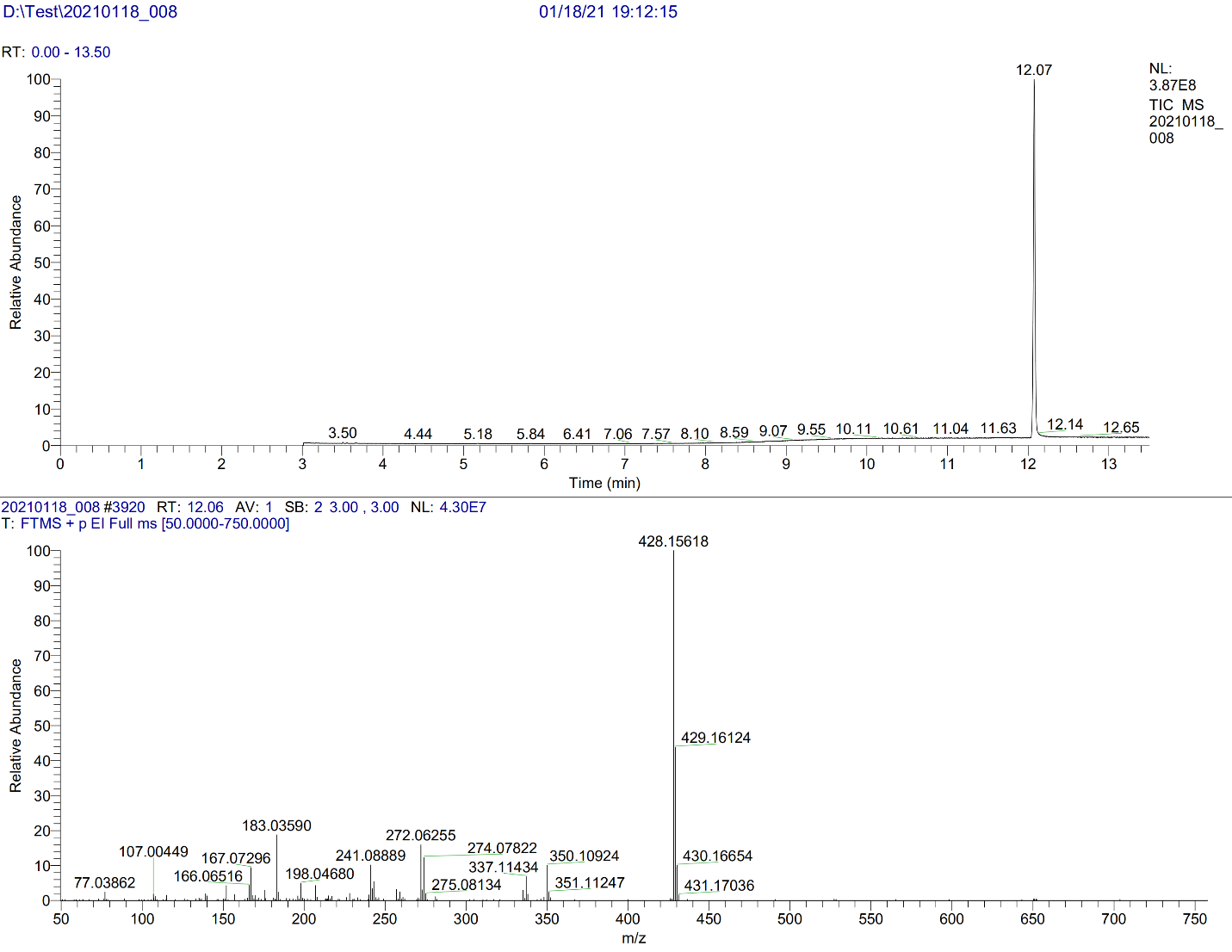


**Figure S31.** High-resolution Mass spectrum of *o*-TATP.


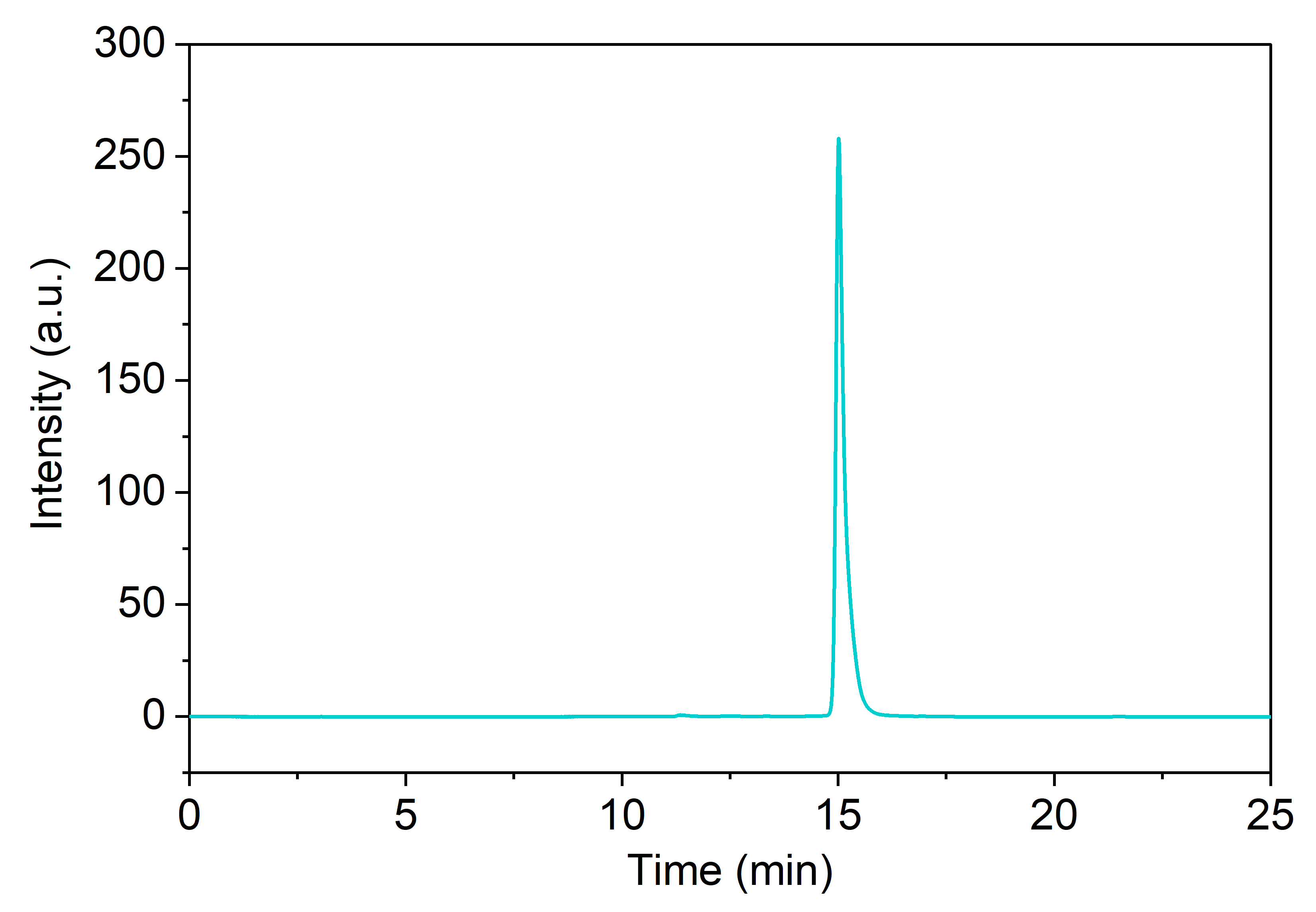


**Figure S32.** HPLC spectrum of *o*-TATP monitored at 254 nm.

**Figure S33.** ^1^H NMR spectrum of *o*-TATPO (in DMSO-*d_6_*).


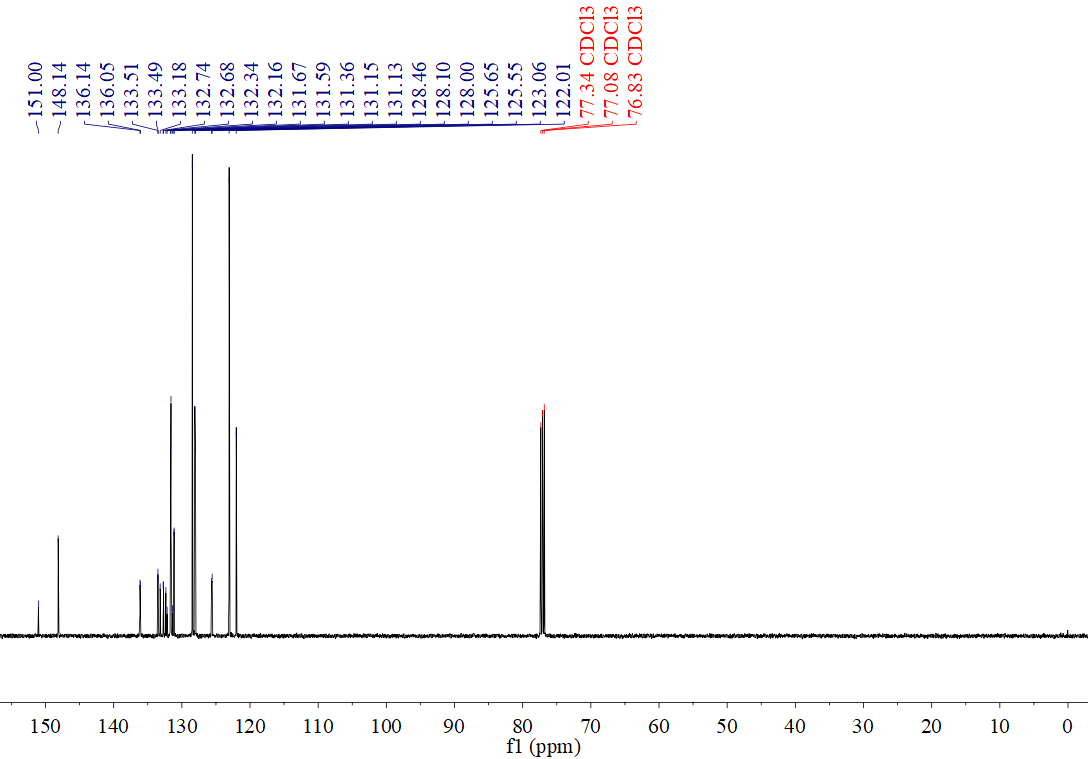


**Figure S34.** ^13^C NMR spectrum of *o*-TATPO (in Chloroform-*d*).


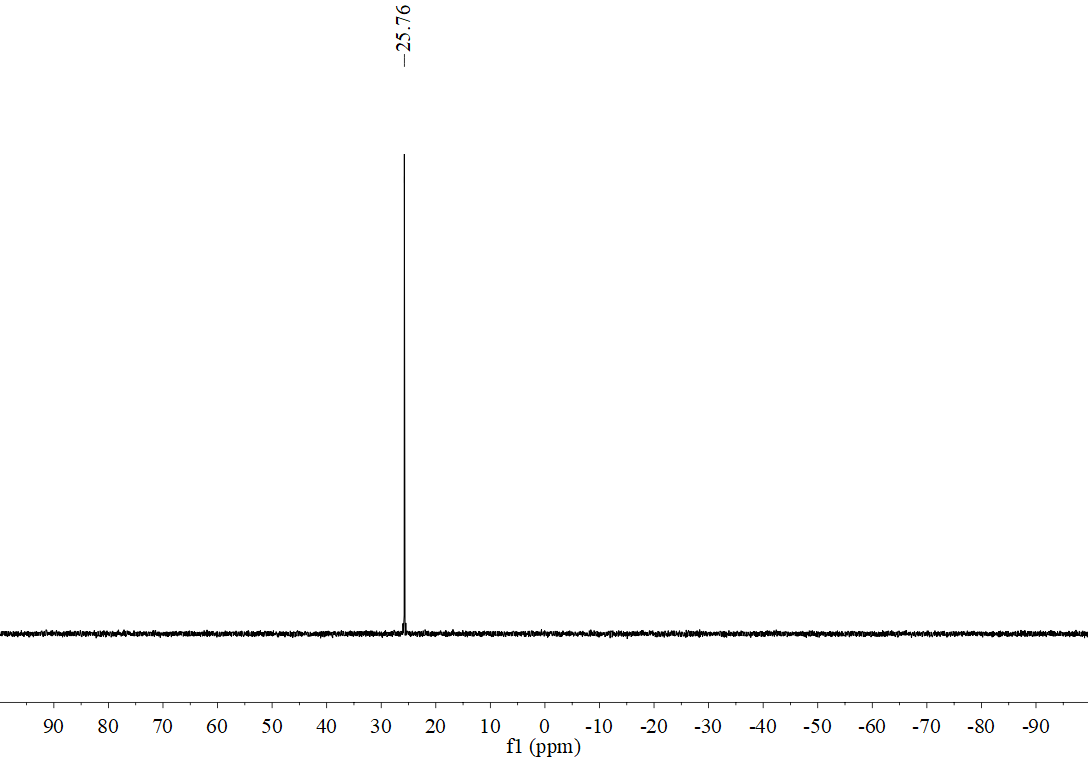


**Figure S35.** ^31^P NMR spectrum of *o*-TATPO (in Chloroform-*d*).


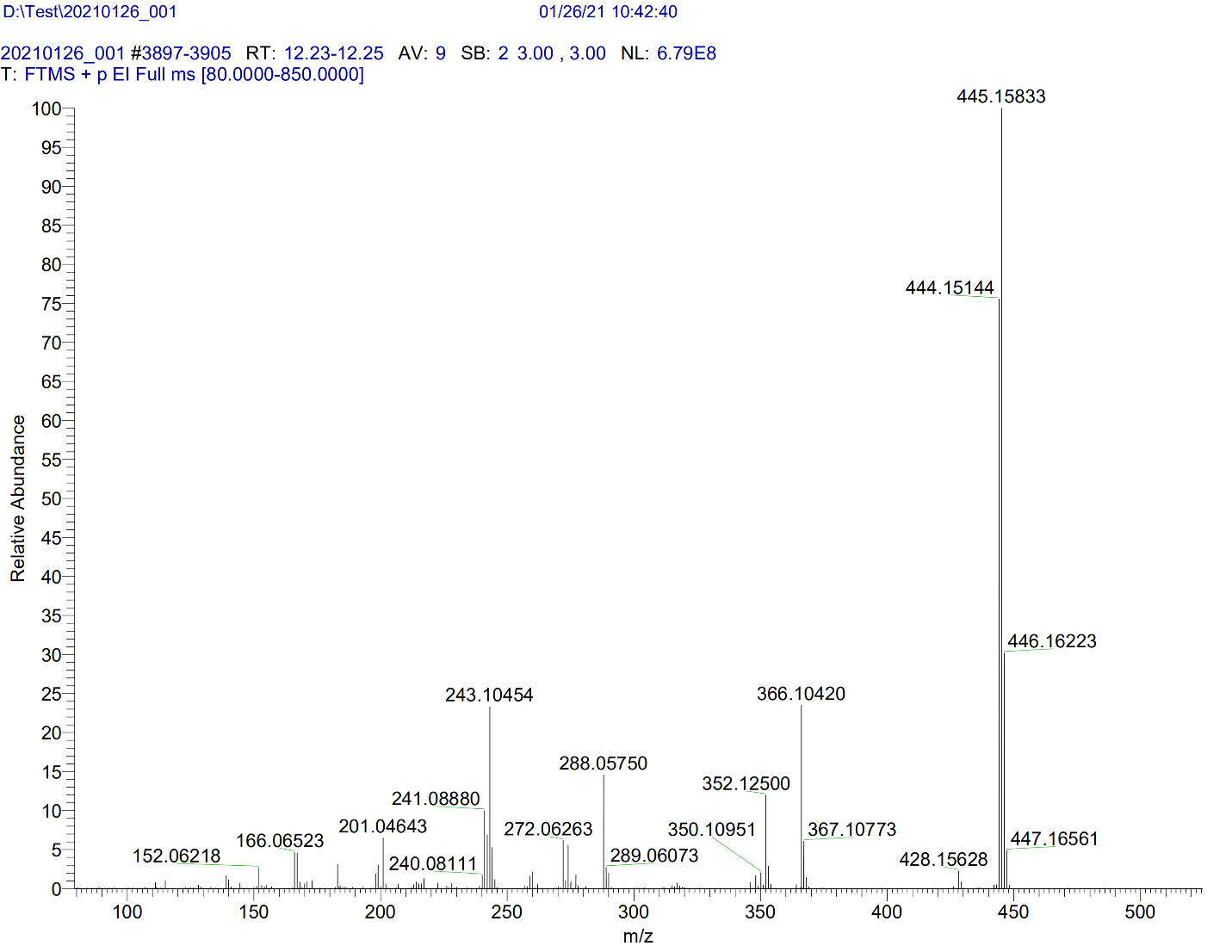


**Figure S36.** High-resolution Mass spectrum of *o*-TATPO.


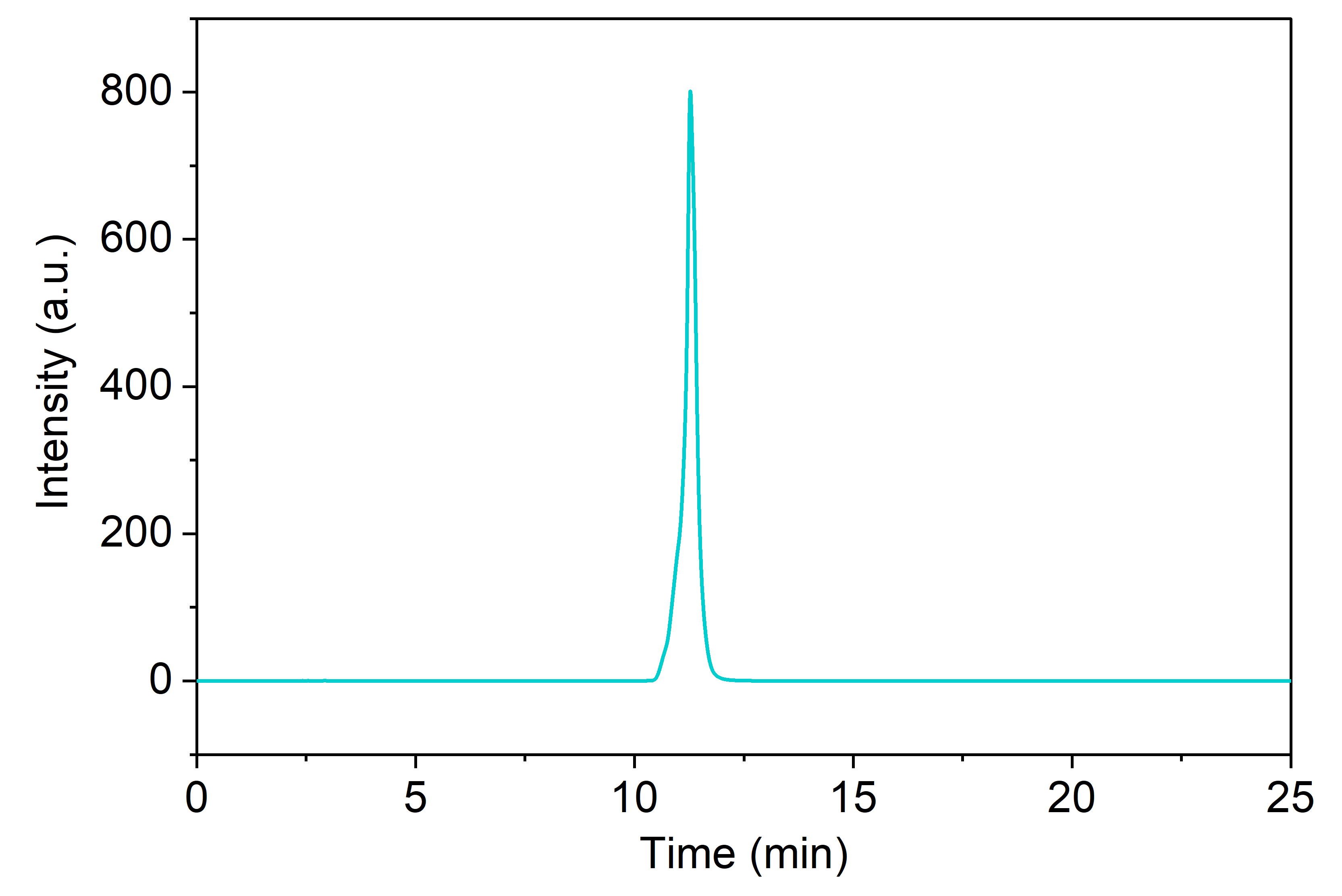


**Figure S37.** HPLC spectrum of *o*-TATPO monitored at 254 nm.

**Figure S38.** ^1^H NMR spectrum of TA (in DMSO-*d_6_*).

**Figure S39.** ^13^C NMR spectrum of TA (in DMSO-*d_6_*).

**Figure S40.** ^1^H NMR spectrum of TPO (in DMSO-*d_6_*).

**Figure S41.** ^13^C NMR spectrum of TPO (in DMSO-*d_6_*).

References

1 Mao, Z. et al. Two-photon-excited ultralong organic room temperature phosphorescence by dual-channel triplet harvesting. *Chemical Science* **10**, 7352-7357 (2019).

2 Yang, Z. et al. Boosting the Quantum Efficiency of Ultralong Organic Phosphorescence up to 52 % via Intramolecular Halogen Bonding. *Angewandte Chemie International Edition* **59**, 17451-17455 (2020).

3 Liu, X. Y. et al. Novel *o*-D-π-A arylamine/arylphosphine oxide hybrid hosts for efficient phosphorescent organic light-emitting diodes. *Organic Electronic* **56**, 186-191 (2018).

4 Wang, L. et al. “Push–pull” 1,8-naphthalic anhydride with multiple triphenylamine groups as electron donor. *J. Mol. Struc.* **1056-1057**, 339-346 (2014).
