# Supplementary material for: Dual-channel mechano-phosphorescence: a combined locking effect with twisted molecular structures and robust interactions
Source: Light Sci Appl. 2024 Apr 8;13:85. doi: 10.1038/s41377-024-01421-5 (PMC11001961; doi:10.1038/s41377-024-01421-5)

checkCIF/PLATON report

Structure factors have been supplied for datablock(s) 211012WHL1_0m

THIS REPORT IS FOR GUIDANCE ONLY. IF USED AS PART OF A REVIEW PROCEDURE FOR PUBLICATION, IT SHOULD NOT REPLACE THE EXPERTISE OF AN EXPERIENCED CRYSTALLOGRAPHIC REFEREE.

No syntax errors found. CIF dictionary Interpreting this report

Datablock: 211012WHL1_0m

Bond precision: C-C = 0.0030 A Wavelength=0.71073

Cell:	a=10.3035(15)	b=10.9790(16)	c=11.2307(17)
	alpha=111.798(2)	beta=102.043(2)	gamma=98.741(2)
Temperature:	100 K		

	Calculated	Reported
Volume	1116.2(3)	1116.2(3)
Space group	P -1	P -1
Hall group	-P 1	-P 1
Moiety formula	C30 H24 N P	C30 H24 N P
Sum formula	C30 H24 N P	C30 H24 N P
Mr	429.47	429.51
Dx,g cm-3	1.278	1.278
Z	2	2
Mu (mm-1)	0.141	0.141
F000	452.0	452.4
F000'	452.35	
h,k,lmax	13,14,14	13,14,14
Nref	5271	4935
Tmin,Tmax	0.935,0.972	0.646,0.746
Tmin'	0.932	

Correction method= # Reported T Limits: Tmin=0.646 Tmax=0.746
AbsCorr = NONE

Data completeness= 0.936 Theta(max)= 27.760

R(reflections)= 0.0486(3915) wR2(reflections)= 0.1337(4935)

S = 1.048 Npar= 289

The following ALERTS were generated. Each ALERT has the format
test-name_ALERT_alert-type_alert-level.
Click on the hyperlinks for more details of the test.

Alert level C

```
PLAT911_ALERT_3_C Missing FCF Refl Between Thmin & STh/L=    0.600         32 Report
PLAT975_ALERT_2_C Check Calcd Resid. Dens.  1.05A   From N1              0.70 eA-3
```

Alert level G

```
PLAT073_ALERT_1_G H-atoms ref, but _hydrogen_treatment Reported as     constr Check
PLAT154_ALERT_1_G The s.u.'s on the Cell Angles are Equal ..(Note)      0.002 Degree
PLAT769_ALERT_4_G CIF Embedded explicitly supplied scattering data     Please Note
PLAT912_ALERT_4_G Missing # of FCF Reflections Above STh/L=  0.600        305 Note
PLAT960_ALERT_3_G Number of Intensities with I  <   - 2*sig(I) ...          6 Check
PLAT978_ALERT_2_G Number C-C Bonds with Positive Residual Density.         14 Info
PLAT982_ALERT_1_G The  P-f'=    0.1043 Deviates from  IT-value =       0.1023 Check
PLAT983_ALERT_1_G The  P-f"=    0.0967 Deviates from  IT-Value =       0.0942 Check
PLAT992_ALERT_5_G Repd & Actual _reflns_number_gt Values Differ by          3 Check
```

0 **ALERT level A** = Most likely a serious problem - resolve or explain
0 **ALERT level B** = A potentially serious problem, consider carefully
2 **ALERT level C** = Check. Ensure it is not caused by an omission or oversight
9 **ALERT level G** = General information/check it is not something unexpected

4 ALERT type 1 CIF construction/syntax error, inconsistent or missing data
2 ALERT type 2 Indicator that the structure model may be wrong or deficient
2 ALERT type 3 Indicator that the structure quality may be low
2 ALERT type 4 Improvement, methodology, query or suggestion
1 ALERT type 5 Informative message, check

It is advisable to attempt to resolve as many as possible of the alerts in all categories. Often the minor alerts point to easily fixed oversights, errors and omissions in your CIF or refinement strategy, so attention to these fine details can be worthwhile. In order to resolve some of the more serious problems it may be necessary to carry out additional measurements or structure refinements. However, the purpose of your study may justify the reported deviations and the more serious of these should normally be commented upon in the discussion or experimental section of a paper or in the "special_details" fields of the CIF. checkCIF was carefully designed to identify outliers and unusual parameters, but every test has its limitations and alerts that are not important in a particular case may appear. Conversely, the absence of alerts does not guarantee there are no aspects of the results needing attention. It is up to the individual to critically assess their own results and, if necessary, seek expert advice.

Publication of your CIF in IUCr journals

A basic structural check has been run on your CIF. These basic checks will be run on all CIFs submitted for publication in IUCr journals (*Acta Crystallographica*, *Journal of Applied Crystallography*, *Journal of Synchrotron Radiation*); however, if you intend to submit to *Acta Crystallographica Section C* or *E* or *IUCrData*, you should make sure that full publication checks are run on the final version of your CIF prior to submission.

Publication of your CIF in other journals

Please refer to the *Notes for Authors* of the relevant journal for any special instructions relating to CIF submission.

PLATON version of 13/07/2021; check.def file version of 13/07/2021

Datablock 211012WHL1_0m - ellipsoid plot

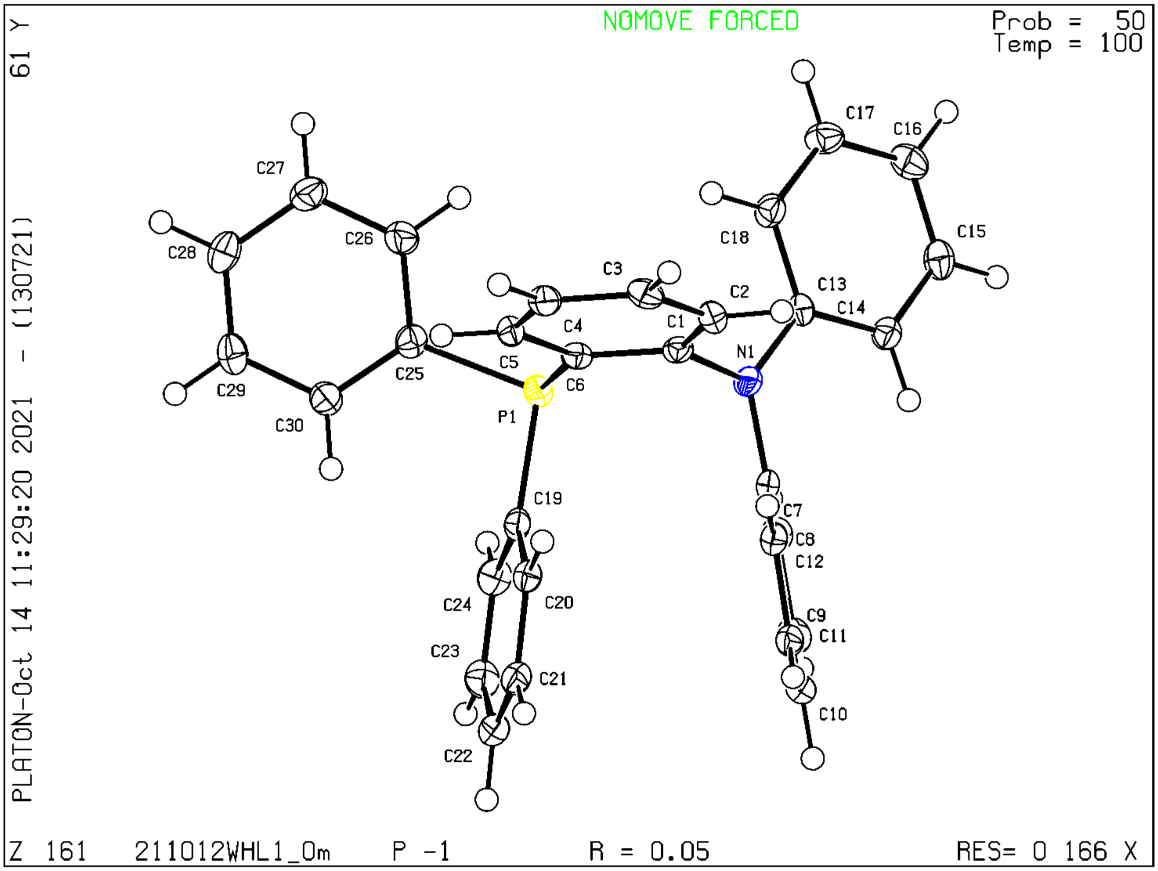

Supplement: Supplementary file 4 — checkCIF_o-TATP [file 41377_2024_1421_MOESM4_ESM.pdf]
