# Supplementary material for: Dual-channel mechano-phosphorescence: a combined locking effect with twisted molecular structures and robust interactions
Source: Light Sci Appl. 2024 Apr 8;13:85. doi: 10.1038/s41377-024-01421-5 (PMC11001961; doi:10.1038/s41377-024-01421-5)
Supplement: Supplementary file 5 — checkCIF_o-TATPO [file 41377_2024_1421_MOESM5_ESM.pdf]

checkCIF (basic structural check) running

Checking for embedded fcf data in CIF ...
Found embedded fcf data in CIF. Extracting fcf data from uploaded CIF, please wait . . . .

checkCIF/PLATON (basic structural check)

Structure factors have been supplied for datablock(s) TPAPO

THIS REPORT IS FOR GUIDANCE ONLY. IF USED AS PART OF A REVIEW PROCEDURE FOR PUBLICATION, IT SHOULD NOT REPLACE THE EXPERTISE OF AN EXPERIENCED CRYSTALLOGRAPHIC REFEREE.

No syntax errors found.
Please wait while processing ....

CIF dictionary
Interpreting this report

Structure factor report

Datablock: TPAPO

Bond precision: C-C = 0.0035 A Wavelength=1.54184

Cell: a=16.0526(6) b=16.6684(6) c=17.1822(7)
alpha=90 beta=90 gamma=90

Temperature: 153 K

	Calculated	Reported
Volume	4597.5(3)	4597.5(3)
Space group	P b c a	P b c a
Hall group	-P 2ac 2ab	-P 2ac 2ab
Moiety formula	C30 H24 N O P	C30 H24 N O P
Sum formula	C30 H24 N O P	C30 H24 N O P
Mr	445.47	445.47
Dx,g cm-3	1.287	1.287
Z	8	8
Mu (mm-1)	1.230	1.230
F000	1872.0	1872.0
F000'	1879.11	
h,k,lmax	19,20,20	19,20,20
Nref	4212	4171
Tmin,Tmax	0.263,0.541	0.701,0.753
Tmin'	0.040	

Correction method= # Reported T Limits: Tmin=0.701 Tmax=0.753 AbsCorr = MULTI-SCAN

Data completeness= 0.990 Theta(max)= 68.295

R(reflections)= 0.0472(3146) wR2(reflections)= 0.1783(4171)

S = 1.092 Npar= 298

The following ALERTS were generated. Each ALERT has the format
test-name_ALERT_alert-type_alert-level.
Click on the hyperlinks for more details of the test.

Alert level B

CRYSS02_ALERT_3_B The value of _exptl_crystal_size_mid is > 0.8
Mid crystal size given = 1.000
CRYSS02_ALERT_3_B The value of _exptl_crystal_size_max is > 1.0
Maximum crystal size given = 2.500

Alert level C

PLAT906_ALERT_3_C Large K Value in the Analysis of Variance ...... 6.342 Check
PLAT911_ALERT_3_C Missing FCF Refl Between Thmin & STh/L= 0.600 40 Report

Alert level G

PLAT063_ALERT_4_G Crystal Size Possibly too Large for Beam Size .. 2.50 mm
PLAT072_ALERT_2_G SHELXL First Parameter in WGHT Unusually Large 0.12 Report

PLAT912_ALERT_4_G Missing # of FCF Reflections Above STh/L= 0.600 2 Note
PLAT978_ALERT_2_G Number C-C Bonds with Positive Residual Density. 2 Info

0 **ALERT level A** = Most likely a serious problem - resolve or explain
2 **ALERT level B** = A potentially serious problem, consider carefully
2 **ALERT level C** = Check. Ensure it is not caused by an omission or oversight
4 **ALERT level G** = General information/check it is not something unexpected

0 ALERT type 1 CIF construction/syntax error, inconsistent or missing data
2 ALERT type 2 Indicator that the structure model may be wrong or deficient
4 ALERT type 3 Indicator that the structure quality may be low
2 ALERT type 4 Improvement, methodology, query or suggestion
0 ALERT type 5 Informative message, check

It is advisable to attempt to resolve as many as possible of the alerts in all categories. Often the minor alerts point to easily fixed oversights, errors and omissions in your CIF or refinement strategy, so attention to these fine details can be worthwhile. In order to resolve some of the more serious problems it may be necessary to carry out additional measurements or structure refinements. However, the purpose of your study may justify the reported deviations and the more serious of these should normally be commented upon in the discussion or experimental section of a paper or in the "special_details" fields of the CIF. checkCIF was carefully designed to identify outliers and unusual parameters, but every test has its limitations and alerts that are not important in a particular case may appear. Conversely, the absence of alerts does not guarantee there are no aspects of the results needing attention. It is up to the individual to critically assess their own results and, if necessary, seek expert advice.

Publication of your CIF in IUCr journals

A basic structural check has been run on your CIF. These basic checks will be run on all CIFs submitted for publication in IUCr journals (*Acta Crystallographica*, *Journal of Applied Crystallography*, *Journal of Synchrotron Radiation*); however, if you intend to submit to *Acta Crystallographica Section C* or *E* or *IUCrData*, you should make sure that full publication checks are run on the final version of your CIF prior to submission.

Publication of your CIF in other journals

Please refer to the *Notes for Authors* of the relevant journal for any special instructions relating to CIF submission.

PLATON version of 18/09/2020; check.def file version of 20/08/2020

Datablock TPAPO - ellipsoid plot

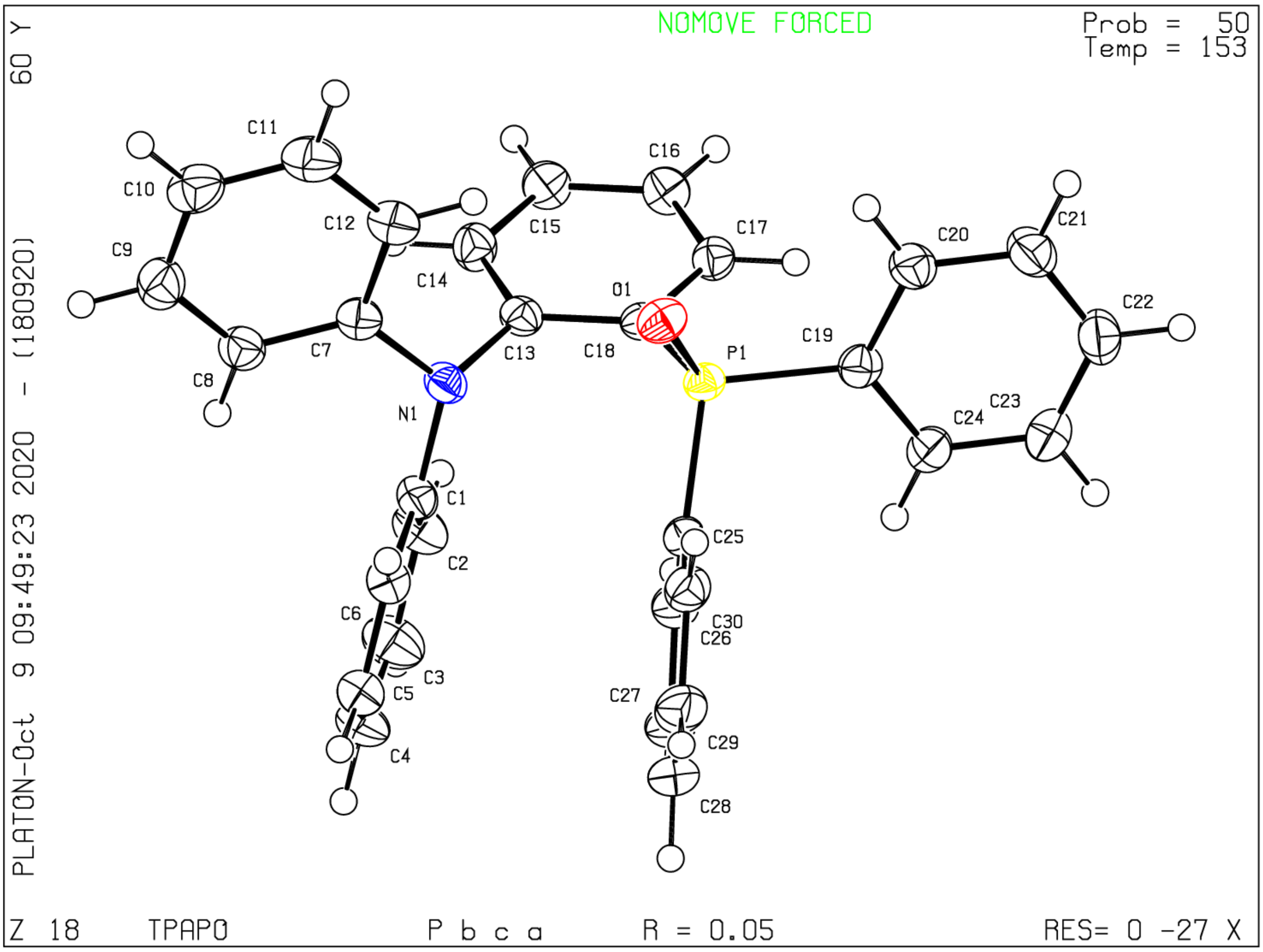

Download CIF editor (publCIF) from the IUCr
Download CIF editor (enCIFer) from the CCDC
Test a new CIF entry
